# Phthalate and gallstones: the mediation of insulin

**DOI:** 10.3389/fpubh.2024.1401420

**Published:** 2024-06-05

**Authors:** Haoxian Tang, Xuan Zhang, Jingtao Huang, Nan Luo, Hongyu Chen, Qinglong Yang, Hanyuan Lin, Hao Hua

**Affiliations:** ^1^Shantou University Medical College, Shantou, Guangdong, China; ^2^Department of Cardiology, The First Affiliated Hospital of Shantou University Medical College, Shantou, Guangdong, China; ^3^Department of Bone and Joint Surgery, Peking University Shenzhen Hospital, Shenzhen, Guangdong, China; ^4^Department of Sports Medicine and Rehabilitation, Peking University Shenzhen Hospital, Shenzhen, Guangdong, China; ^5^Department of Psychiatry, Shantou University Mental Health Center, Shantou, Guangdong, China; ^6^Department of Urology, The Second Affiliated Hospital of Shantou University Medical College, Shantou, Guangdong, China; ^7^Department of Hepatic-Biliary-Pancreatic Surgery, The Affiliate Hospital of Guizhou Medical University, Guiyang, Guizhou, China

**Keywords:** plasticizer, cholelithiasis, hyperinsulinemia, mediation, NHANES

## Abstract

**Background:**

Exposure to a mixture of environmental chemicals may cause gallstone, but the evidence remains equivocal. The current study aims to investigate the association between phthalate metabolites and gallstones, and to explore their mediators.

**Methods:**

Data from the National Health and Nutrition Examination Survey 2017–2018 on U.S. adults (≥20 years) were analyzed to explore the association between phthalate metabolites and gallstones by employed survey-weighted logistic regression, restricted cubic spline (RCS), weighted quantile sum (WQS) regression, and Bayesian kernel machine regression (BKMR). Mediation analyses examined the role of oxidative stress markers, inflammatory markers, metabolic syndrome, body composition, diabetes, and insulin.

**Results:**

The current study included 1,384 participants, representing 200.6 million U.S. adults. Our results indicated a significant association between phthalate metabolites, particularly high molecular weight metabolites such as Di(2-ethylhexyl) phthalate (DEHP) and 1,2-Cyclohexane dicarboxylic acid diisononyl ester (DINCH), and gallstones. Furthermore, mediation analyses indicated that phthalate metabolites may play a role in the development of gallstones by influencing insulin secretion. Subgroup analyses did not reveal significant interaction.

**Conclusion:**

The association between exposure to phthalates and the occurrence of gallstones, potentially mediated by hyperinsulinemia from a nationally representative epidemiological perspective. These insights contribute to a better understanding of the potential health implications of plasticizers, emphasizing the need for proactive management measures.

## Introduction

Gallstones, arising from heightened levels of cholesterol or bilirubin in bile, manifest as crystalline deposits within the gallbladder, categorized by their composition as cholesterol stones, pigment stones, or a combination of both, and play a pivotal role in the etiology of gallbladder-related conditions (1, 2). The higher incidence of hospital admissions linked to gallstone prevalence, compared to other gastroenterological conditions, poses risks like cholecystitis, pancreatitis, biliary tract obstruction, and gallbladder cancer (3, 4). Survey data suggests that around 20 to 25 million Americans are affected by gallstones or are expected to develop them, impacting 10 to 15% of the adult population. This prevalence results in a significant economic burden, with an estimated cost of approximately $4 billion per year (5, 6). Risk factors for gallstones include pregnancy, physical inactivity, obesity, over nutrition, a high waist-to-height ratio, high blood pressure, and a history of hypertension or familial gallstones (1, 7). However, the etiology of gallstones remains incompletely understood. Although previous studies have investigated the impact of specific metal exposure on the development of gallstones (8), there are still other environmental factors that warrant further exploration.

Phthalates, a versatile class of chemicals exhibiting endocrine-disrupting properties, represent the predominant category of global plasticizers, finding extensive use in cosmetics, personal care products, plastics, and construction materials. Due to their lack of chemical binding to products, phthalates are released into the environment, potentially entering the body through ingestion, inhalation, and dermal absorption (9, 10). Statistically, the daily intake of phthalates through all routes varies between 0.08 and 69.58 ug/kg (11). Exposure to phthalates has been correlated with various human diseases, including asthma, allergies, and reproductive and developmental disorders (12, 13). About 90% of phthalates are used in pvc production and these are also the main culprits for aggressive malignant diseases such as thyroid angiosarcoma (14, 15). Crucially, the consumption of phthalate products, which witnessed a 21% increase between 2014 and 2019 (16), has gradually emerged as a significant public health concern. Their endocrine-disrupting characteristics can lead to interference with different cellular signaling pathways involved in body weight and glucose homeostasis (17), playing a non-negligible role in the development of gallstones. Nevertheless, the association between phthalates and gallstones, along with their potential mechanisms of action in the pathogenesis of gallstones, remains largely unexplored.

Insulin, synthesized by pancreatic β cells, maintains normal blood glucose levels by promoting glucose uptake and metabolism while inhibiting processes like glycogenolysis, gluconeogenesis, and triglyceride breakdown (18). Animal experiments have demonstrated that phthalates modulate the mitochondrial apoptotic pathway and induce oxidative stress, possibly by activating peroxisome proliferator-activated receptors (PPARs). This action may interfere with the post-receptorial action of insulin, leading to a state of insulin resistance (19, 20). Concurrently, epidemiological studies have indicated that hyperinsulinemia is associated with an increased predisposition to gallstones (21, 22). Therefore, considering the pivotal role of insulin in the context of phthalates and gallstones, we hypothesize that phthalates may elevate the risk of gallstones by inducing hyperinsulinemia.

In summary, there is a necessity for an epidemiological study to evaluate the associations between phthalate and gallstones. Simultaneously, we also targeted to explore the mediating effect of insulin. This study aimed to investigate this association through the analysis of cross-sectional data obtained from the National Health and Nutrition Examination Survey (NHANES), encompassing a national representative sample.

## Methods

### Data sources

The NHANES is a series of surveys conducted by the National Center for Health Statistics (NCHS), employing a sophisticated multistage probability cluster design to assess the health and nutritional well-being of a representative sample from the non-institutionalized US population. Comprehensive details are accessible in the NHANES survey methods and analytic guidelines (23). Extracted data originated from the 2017–2018 NHANES cycles, specifically focusing on participants aged 20 years and above. The data underwent analysis between September and December 2023. Ethical approval for NHANES procedures and protocols was obtained from the NCHS Research Ethics Review Board, and all participants provided written informed consent. The study followed the reporting guidelines set forth by the Strengthening the Reporting of Observational Studies in Epidemiology.

### Study design and population

The current study included 2,986 participants from the NHANES 2017–2018 cycle who underwent urinary phthalate metabolite measurements. Exclusion criteria were applied post-data collection, removing participants <20 years old (*n* = 1,221), those with incomplete data on urinary phthalates metabolite and gallstones (*n* = 65), and individuals lacking demographic information (*n* = 230), including age, sex, race/ethnicity, marital status, poverty income ratio (PIR), and educational level. Additional exclusions were made for those without data on physical activity (PA), drinking status, smoking status, body mass index (BMI), and urinary creatinine (*n* = 86). The final dataset for analysis comprised 1,384 participants (Supplementary Figure S1).

### Assessment of gallstones

In NHANES, the presence of gallstones was evaluated through the question by asking people over the age of 20, “Has a doctor or other health professional ever told you that you had gallstones?.” A response of “yes” to this question was considered indicative of gallstones, while an exact response of “no” was categorized as the absence of gallstones, as previously mentioned in the literature (24). The occurrence of gallstones served as the outcome variable for analysis.

### Phthalates metabolite measurement

This study investigated 19 urinary phthalate metabolites, as outlined in Supplementary Table S1, including Mono (3-carboxypropyl) phthalate (MCPP), Mono-ethyl phthalate (MEP), Mono-isobutyl phthalate (MiBP), Mono-2-hydroxy-iso-butyl phthalate (MHiBP), Mono-n-butyl phthalate (MBP), Mono-3-hydroxybutyl phthalate (MHBP), Monobenzyl phthalate (MBzP), Mono(2-ethylhexyl) phthalate (MEHP), Mono(2-ethyl-5-carboxypentyl) phthalate (MECPP), Mono(2-ethyl-5-hydroxyhexyl) phthalate (MEHHP), Mono(2-ethyl-5-oxohexyl) phthalate (MEOHP), Mono-isononyl phthalate (MNP), Monocarboxyisooctyl phthalate (MCOP), Mono-oxo-isononyl phthalate (MONP), Monocarboxy-isononyl phthalate (MCNP), Cyclohexane-1,2-dicarboxylic acid mono(hydroxy-isononyl) ester (MHINCH), Cyclohexane-1,2-dicarboxylic acid mono(carboxyoctyl) ester (MCOCH), Mono(2-ethyl-5-hydroxyhexyl) terephthalate (MEHHTP), and Mono(2-ethyl-5-carboxypentyl) terephthalate (MECPTP). Among them, MNP is excluded from the analysis due to over 80.07% of participants having concentrations below the limit of detection (LOD) to mitigate potential bias. The LOD and molecular weight (MW) of each metabolite and its corresponding parent compound are shown in Supplementary Table S1.

The quantification of phthalate metabolites in urine employed a high-performance liquid chromatography-electrospray ionization-tandem mass spectrometry (HPLC-ESI-MS/MS) method (25). This process involves enzymatic deconjugation of glucuronidated phthalate monoesters in urine samples, followed by on-line solid phase extraction (SPE) coupled with reversed phase HPLC-ESI-MS/MS. The use of isotopically-labeled internal standards for the phthalate metabolites improves assay precision. In addition, 4-methyl umbelliferone glucuro- nide is employed to monitor deconjugation efficiency. This method enables the rapid and selective detection of monoester metabolites of commonly used phthalate diesters in human urine, with LOD in the low ng/mL range. Concentrations below the LOD were replaced using the LOD/√2 method (26).

Sum phthalate metabolites were calculated to assess the influence of the parent chemical, including Di(2-ethylhexyl) phthalate (ΣDEHP, expressed as MEHP, MW = 278.2 g/mol), Di-isobutyl phthalate (ΣDiBP, expressed as MiBP, MW = 222.2 g/mol), Di-n-butyl phthalate (ΣDBP, expressed as MBP, MW = 222.2 g/mol), Di-isononyl phthalate (ΣDNP, expressed as MONP, MW = 306.4 g/mol), 1,2-Cyclohexane dicarboxylic acid diisononyl ester (ΣDINCH, expressed as MHINCH, MW = 314.4 g/mol), and Di(2-ethylhexyl) terephthalate (ΣDEHTP, expressed as MEHP, MW = 294.3 g/mol). High MW (> 250) phthalate (high-MWP) is the molar sum of MCPP, MBzP, MHiBP, MEHP, MECPP, MEHHP, MEOHP, MCOP, MONP, MCNP, MHINCH, MCOCH, MEHHTP, and MECPTP (expressed as MCPP, MW = 252.2). Low MW (< 250) phthalate (low-MWP) is the molar sum of MEP, MiBP, MHiBP, MBP, and MHBP (expressed as MEP, MW = 194.2) (27).

### Covariates

Several potential confounding variables were considered based on published research and clinical judgment, including age, sex, marital status, race/ethnicity, education level, PIR, BMI, PA, smoking status, drinking status, and creatinine (28). Age, PIR, BMI, PA, and creatinine were included in the adjustment model as continuous variables. In the subgroup analysis, age was categorized as <40, 40–59, and ≥ 60 when utilized as an exposure variable or considered in subgroup analyses. Sex was categorized into female and male. Marital status was grouped into married, never married, living with a partner, and other (e.g., widowed, divorced, or separated). Self-reported race/ethnicity was categorized as Mexican American, non-Hispanic White, non-Hispanic Black, other Hispanic, and others (including multi-racial participants). Educational level was classified as less than high school, high school or equivalent, and above high school. BMI was calculated as the weight (in kilograms) divided by the square of the height (in meters). PA was defined as the time individuals reported spending during the week on activities such as walking or biking, tasks around the home or yard, work, and recreational activity (29). Smoking status was characterized as never (<100 cigarettes smoked in life), former (>100 cigarettes smoked but currently quit), and now (>100 cigarettes smoked and currently smoking) (30). Drinking status was categorized as never (<12 drinks lifetime), former (<12 drinks lifetime or none in the past year), mild (≤1 drink per day for females and ≤ 2 drinks per day for males in the past year), moderate (≤2 drinks per day for females and ≤ 3 drinks per day for males in the past year), and heavy (≥3 drinks per day for females and ≥ 4 drinks per day for males in the past year) (31). Creatinine was measured from the laboratory.

### Statistical analysis

Complex sampling design and environmental subsample weights were considered in our analyses. Participant characteristics were calculated based on the presence or absence of gallstone, with categorical variables presented as numbers and percentages, and continuous variables as means and standard error (SE). Differences across groups were analyzed using the chi-squared test with Rao & Scott’s second-order correction (for categorical variables) and the Wilcoxon rank-sum test (for continuous variables).

The concentrations of individual and sums of phthalate metabolite were naturally log-transformed to achieve a normal distribution, and they were divided into three groups (Tertile1-Tertile3) according to percentile. Survey-weighted multivariable logistic regression was performed to assess associations of individuals and sums phthalate metabolite (treated as both a categorical and continuous variable) with gallstone. The crude model did not account for covariates, while the adjusted model accounted for age, sex, race/ethnicity, PIR, marital status, education level, BMI, PA, smoking status, drinking status, and creatinine. Possible nonlinear effects were modeled using restricted cubic spline (RCS) models with 3 knots at 10, 50, and 90%.

Further to calculating the sum of phthalate metabolites based on MW, we employed both weighted quantile sum (WQS) regression and Bayesian kernel machine regression (BKMR) to analyze the joint effects of phthalate metabolites. The WQS regression model built a weighted index to estimate the combined effects of all predictive factors related to the outcomes, demonstrating high sensitivity and specificity in identifying significant exposures (32). Bootstrapping with 1,000 iterations was employed to construct the WQS index as a representative measure of mixed exposure levels to phthalates, and the contribution weights of each compound to the overall impact of the mixture were calculated. The dataset was randomly divided, with 40% allocated to the training set and the remaining 60% used as the validation set. The WQS model estimated the incremental risk of gallstones corresponding to a 25% (one-quarter) increase in the WQS index. BKMR is an approach that models the health effects of complex chemical mixtures, using flexible functions and Bayesian methods to identify important mixture components and account for correlations, particularly in high-dimensional settings (33). After adjusting for all covariates, this model underwent 10,000 iterations using the Markov Chain Monte Carlo algorithm. Posterior incorporation probabilities (PIPs), spanning from 0 to 1, were derived from the BKMR model to ascertain the relative significance of each exposure concerning the outcome, with a defined threshold of 0.5.

In additionally analysis, we further explored potential mediators of the association between phthalate exposure and gallstone, including oxidative stress markers (including bilirubin, uric acid, and gamma glutamyl transferase) (34), inflammatory markers (including C-reactive protein, alkaline phosphatase, neutrophils, and ferritin) (35), metabolic syndrome (36), body composition [including BMI, waist circumference, visceral adiposity index (37), lean/fat mass measured using dual-energy X-ray absorptiometry (38)], diabetes and insulin resistance (including fasting blood glucose [FBG], fasting serum insulin [FINS], triglyceride glucose index, and insulin resistance [HOMA-IR], insulin sensitivity [HOMA-IS], and β-cell function [HOMA-β] assessed through the homeostasis model assessment [HOMA]) (39, 40). Mediation analyses were conducted by using the Sobel test, Bootstrap, and the quasi-Bayesian Monte Carlo method with 1,000 simulations based on normal approximation (41, 42). Stratified analyses were performed based on age (<40, 40–59, or ≥ 60 years), sex (female or male), race/ethnicity (Mexican American, non-Hispanic Black, non-Hispanic White, other Hispanic, or other), and BMI (<25, 25–29, ≥30). The interaction was assessed by adding a cross-product term to the regression model and calculating the *p* value using the likelihood ratio test, comparing models with and without the interaction term.

All analyses were conducted with R (4.2.3) and Free Software Foundation statistics software (version 1.9.2). Statistical significance was determined by two-sided *p* values below 0.05.

## Results

### Characteristics of the participants

Table 1 presents the characteristics of a sample representing 200.6 million U.S. adults for analysis. The weighted mean age was 47.45 years, with females comprising 50.94%. Among this population, 21.20 million had gallstones, more prevalent among those aged ≥60 years (weighted percentage, 50.45%) and women (weighted percentage, 75.76%). Compared to those without gallstones, individuals with gallstones had higher BMI, HOMA-IR, HOMA-β, fasting insulin, MECPP, MEOHP, MCOP, and ΣDEHP levels, alongside lower HOMA-IS and MCPP concentrations.

**Table 1:** Characteristics of study population, NHANES 2017–2018.

Characteristics	Total	Without gallstone	With gallstone	*p* value
Weighted population, *n* [in millions]	200.06	178.85	21.20	
Age, mean (SE), years	47.45 (0.83)	46.27 (0.94)	57.38 (1.40)	< 0.001
Age, *n* [in millions] (%)				<0.001
<40	74.77 (37.37)	71.95 (40.23)	2.81 (13.26)	
40–59	70.63 (35.30)	62.93 (35.19)	7.69 (36.29)	
≥60	54.66 (27.32)	43.97 (24.58)	10.70 (50.45)	
Sex, *n* [in millions] (%)				< 0.001
Female	101.92 (50.94)	85.85 (48.00)	16.06 (75.76)	
Male	98.14 (49.06)	93.00 (52.00)	5.14 (24.24)	
Race/ethnicity, *n* [in millions] (%)				0.19
Mexican American	16.90 (8.45)	15.18 (8.49)	1.72 (8.09)	
Non-Hispanic Black	21.74 (10.86)	19.86 (11.10)	1.88 (8.86)	
Non-Hispanic White	129.74 (64.85)	114.23 (63.87)	15.51 (73.13)	
Other Hispanic	12.43 (6.21)	11.88 (6.64)	0.55 (2.61)	
Other race	19.25 (9.62)	17.71 (9.90)	1.55 (7.30)	
Marital status, *n* [in millions] (%)				0.04
Married	105.02 (52.50)	94.89 (53.05)	10.13 (47.78)	
Never married	41.33 (20.66)	38.77 (21.68)	2.56 (12.07)	
Living with partner	17.52 (8.76)	15.55 (8.69)	1.97 (9.31)	
Other	36.18 (18.09)	29.64 (16.57)	6.54 (30.84)	
Educational level, *n* [in millions] (%)				0.76
Less than high school	19.84 (9.92)	17.30 (9.67)	2.54 (11.98)	
High school or equivalent	53.56 (26.77)	47.72 (26.71)	5.79 (27.29)	
Above high school	126.66 (63.31)	113.78 (63.62)	128.77 (60.73)	
PIR, mean (SE)	3.13 (0.07)	3.17 (0.08)	2.81 (0.14)	0.09
BMI, mean (SE), kg/m^2^	29.75 (0.32)	29.31 (0.27)	33.44 (0.70)	< 0.001
Physical activity, mean (SE), min/week	1100.17 (71.13)	1125.96 (72.58)	882.61 (167.61)	0.25
Smoking status, *n* [in millions] (%)				0.045
Never	123.01 (61.49)	112.94 (63.15)	10.06 (47.46)	
Former	43.86 (21.93)	37.65 (21.05)	6.22 (29.31)	
Now	33.19 (16.59)	28.26 (15.80)	4.93 (23.23)	
Drinking status, *n* [in millions] (%)				0.16
Never	12.23 (6.11)	10.64 (5.95)	1.60 (7.53)	
Former	27.68 (13.84)	23.06 (12.89)	4.62 (21.79)	
Now	160.15 (80.05)	145.16 (81.16)	14.99 (70.68)	
Creatinine, mean (SE), mg/dl	124.78 (3.83)	125.27 (3.86)	120.65 (7.82)	0.80
**HOMA**				
HOMA-IR, mean (SE)	3.51 (0.22)	3.21 (0.18)	5.57 (0.86)	<0.001
HOMA-IS, mean (SE)	0.56 (0.03)	0.59 (0.03)	0.33 (0.04)	< 0.001
HOMA-β, mean (SE)	101.51 (5.33)	97.53 (4.42)	128.65 (13.43)	0.01
Fasting insulin, mean (SE), μU/mL	12.08 (0.60)	11.34 (0.52)	17.13 (1.92)	<0.001
**Individual phthalate, mean (SE), ng/mL**				
MEP	141.08 (34.52)	143.31 (38.44)	122.31 (37.57)	0.65
MiBP	12.17 (1.02)	12.05 (1.01)	13.19 (3.38)	0.61
MHiBP	3.67 (0.30)	3.63 (0.28)	4.00 (1.07)	0.45
MBP	13.18 (0.67)	12.87 (0.66)	15.82 (2.39)	0.17
MHBP	1.19 (0.07)	1.17 (0.08)	1.28 (0.20)	0.32
MCPP	3.06 (1.14)	3.18 (1.27)	2.09 (0.27)	0.02
MBzP	6.65 (0.37)	6.31 (0.37)	9.51 (3.30)	0.16
MEHP	1.53 (0.07)	1.58 (0.08)	1.11 (0.09)	0.08
MECPP	10.68 (0.63)	10.41 (0.66)	12.97 (2.10)	0.004
MEHHP	7.05 (0.39)	6.99 (0.42)	7.54 (1.18)	0.10
MEOHP	4.56 (0.24)	4.53 (0.26)	4.84 (0.66)	0.03
MCOP	9.85 (0.79)	9.82 (0.87)	10.10 (2.11)	0.05
MONP	2.82 (0.24)	2.80 (0.25)	3.03 (0.96)	0.21
MCNP	2.23 (0.29)	2.28 (0.32)	1.76 (0.09)	0.11
MHINCH	2.47 (0.22)	1.95 (0.23)	6.83 (1.59)	0.96
MCOCH	1.51 (0.14)	1.20 (0.13)	4.15 (0.92)	0.24
MEHHTP	26.75 (4.06)	25.91 (4.48)	33.85 (12.95)	0.74
MECPTP	97.51 (14.89)	99.64 (16.55)	79.56 (25.11)	0.17
**Sums phthalate, mean (SE), ng/mL**				
Low-MWP	168.03 (34.36)	169.86 (38.17)	152.65 (42.77)	0.60
High-MWP	147.25 (17.08)	147.14 (18.34)	148.17 (34.36)	0.89
ΣDNP	12.18 (0.95)	12.13 (1.05)	12.63 (2.96)	0.08
ΣDBP	14.29 (0.73)	13.97 (0.72)	17.02 (2.58)	0.18
ΣDiBP	15.61 (1.29)	15.46 (1.27)	16.93 (4.37)	0.82
ΣDEHP	22.18 (1.18)	21.90 (1.26)	24.55 (3.59)	0.02
ΣDINCH	3.92 (0.35)	3.10 (0.35)	10.80 (2.34)	0.77
ΣDEHTP	119.83 (17.89)	121.02 (19.92)	109.80 (36.36)	0.26

All means and SEs for continuous variables and numbers and percentages for categorical variables were weighted. BMI, body mass index; DBP, di-n-butyl phthalate; DEHP, di(2-ethylhexyl) phthalate; DEHTP, di(2-ethylhexyl) terephthalate; DiBP, di-isobutyl phthalate; DINCH, 1,2-Cyclohexane dicarboxylic acid, diisononyl ester; DNP, di-isononyl phthalate; High − MWP, high molecular-weight phthalate; Low − MWP, low molecular-weight phthalate; HOMA, the homeostasis model assessment; HOMA-IR, homeostatic model assessment of insulin resistance; HOMA-IS, homeostasis model assessment of insulin sensitivity; HOMA-β, homeostasis model assessment of β-cell function; MBP, mono-n-butyl phthalate; MBzP, monobenzyl phthalate; MCNP, monocarboxy-isononyl phthalate; MCOCH, cyclohexane-1,2-dicarboxylic acid mono(carboxyoctyl) ester; MCOP, monocarboxyisooctyl phthalate; MCPP, mono (3-carboxypropyl) phthalate; MECPP, mono(2-ethyl-5-carboxypentyl) phthalate; MECPTP, mono-2-ethyl-5-carboxypentyl terephthalate; MEHP, mono(2-ethyl-5-carboxypentyl) phthalate; MEHHP, mono(2-ethyl-5-hydroxyhexyl) phthalate; MEHHTP, mono(2-ethyl-5-hydroxyhexyl) terephthalate; MEOHP, mono-(2-ethyl-5-oxohexyl) phthalate; MEP, mono-ethyl phthalate; MHBP, mono-3-hydroxybutyl phthalate; MHiBPP, mono-2-hydroxy-iso-butyl phthalate; MHINCH, cyclohexane-1,2-dicarboxylic acid mono(hydroxy-isononyl) ester; MiBP, Mono-isobutyl phthalate; MONP, mono-oxo-isononyl phthalate; PIR, poverty income ratio; SE, standard error.

### Association between phthalate metabolite and gallstones

**Figure 1:**
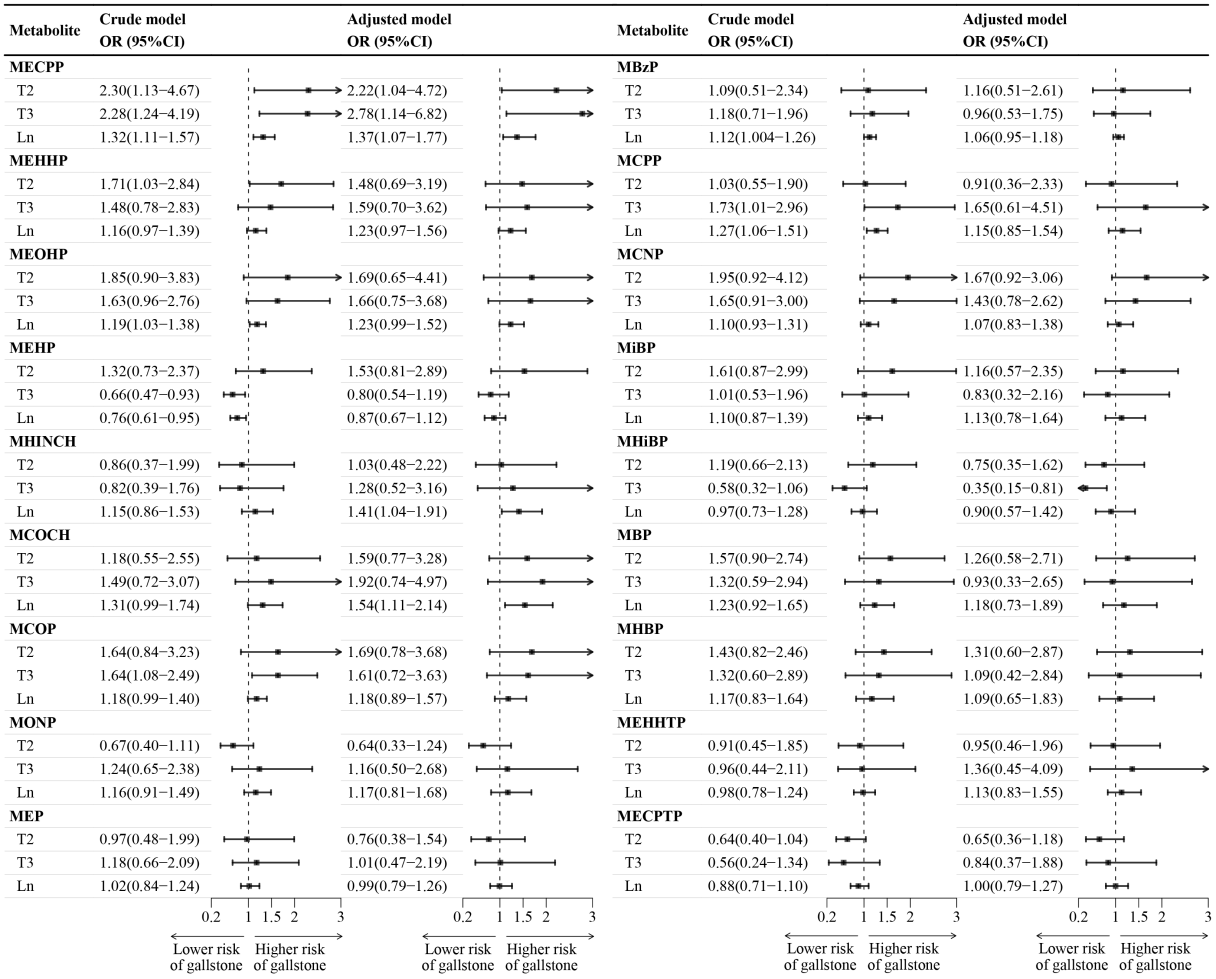
Association between individual phthalate metabolite and gallstone. CI, confidence interval; Ln, natural logarithm; MBP, mono-n-butyl phthalate (ng/mL); MBzP, monobenzyl phthalate; MCNP, monocarboxy-isononyl phthalate; MCOCH, cyclohexane-1,2-dicarboxylic acid mono(carboxyoctyl) ester; MCOP, monocarboxyisooctyl phthalate; MCPP, mono (3-carboxypropyl) phthalate; MECPP, mono(2-ethyl-5-carboxypentyl) phthalate; MECPTP, mono-2-ethyl-5-carboxypentyl terephthalate; MEHP, mono(2-ethyl-5-carboxypentyl) phthalate; MEHHP, mono(2-ethyl-5-hydroxyhexyl) phthalate; MEHHTP, mono(2-ethyl-5-hydroxyhexyl) terephthalate; MEOHP, mono-(2-ethyl-5-oxohexyl) phthalate; MEP, mono-ethyl phthalate; MHBP, mono-3-hydroxybutyl phthalate; MHiBPP, mono-2-hydroxy-iso-butyl phthalate; MHINCH, cyclohexane-1,2-dicarboxylic acid mono(hydroxy-isononyl) ester; MiBP, Mono-isobutyl phthalate; MONP, mono-oxo-isononyl phthalate; OR, odds ratios; T2, second tertile; T3, third tertile. ^a^Tertile 1 is the reference category. ^b^The crude model did not account for covariates, while the adjusted model accounted for age, sex, race/ethnicity, poverty income ratio, marital status, education level, body mass index, physical activity, smoking and drinking status, and creatinine.

Figure 1 illustrated the association between individual phthalate metabolites and gallstones. In the adjusted multivariable logistic regression, each one-unit increase in the natural log-transformed concentrations of MECPP, MHINCH, and MCOCH was associated with a higher incidence of gallstones by 37, 41, and 54%, respectively. Individuals in the third tertile exhibited a lower incidence of gallstones compared to those with MHiBP concentrations in the first tertile. Higher concentrations of MEOHP, MBzP, MCOP, and MCPP were associated with a higher incidence of gallstones in the crude model, but this association was no longer observed after accounting for confounding factors.

**Figure 2:**
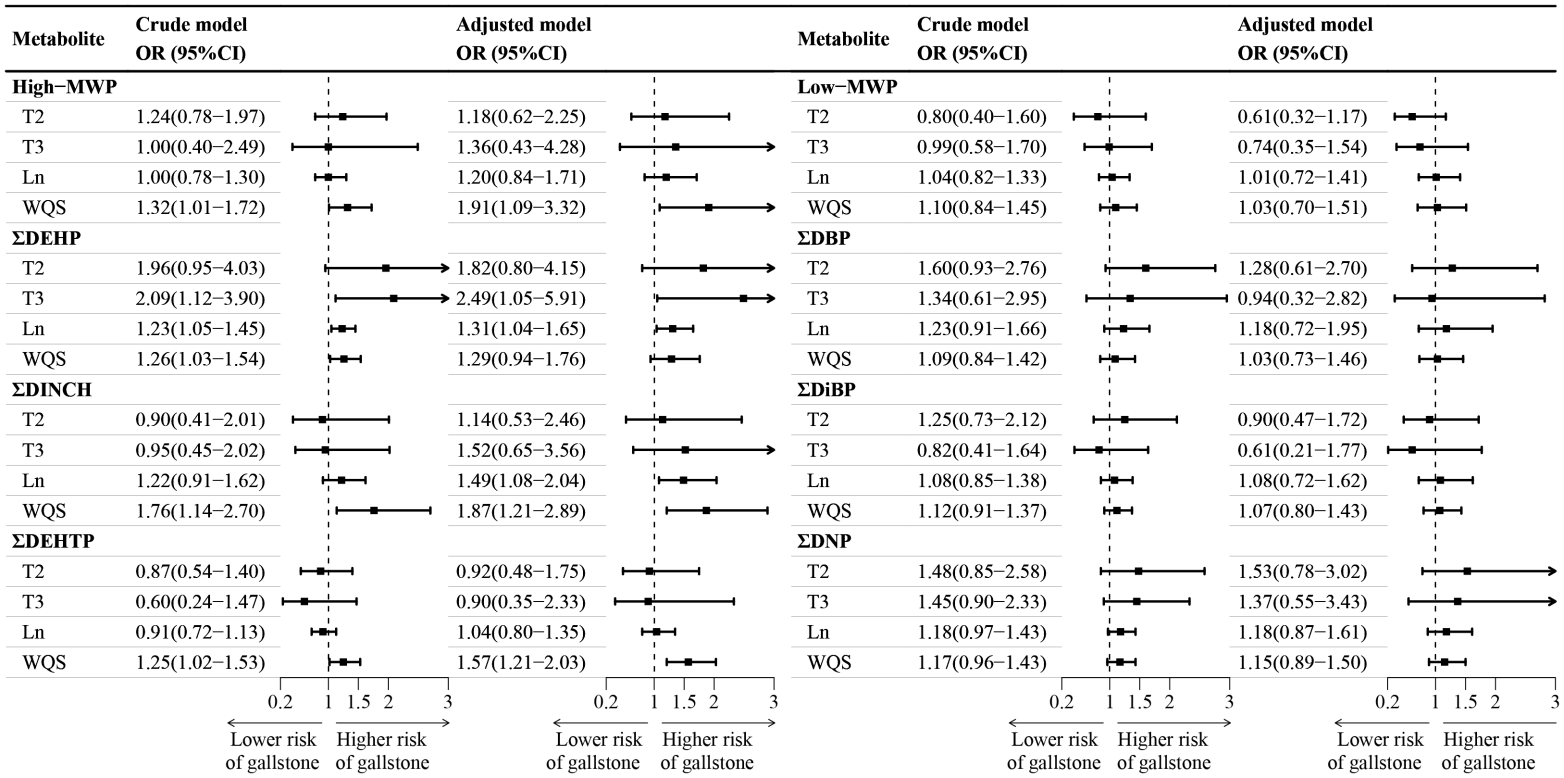
Association between sums phthalate metabolite and gallstone. CI, confidence interval; DBP, di-n-butyl phthalate; DEHP, di(2-ethylhexyl) phthalate; DEHTP, di(2-ethylhexyl) terephthalate; DiBP, di-isobutyl phthalate; DINCH, 1,2-Cyclohexane dicarboxylic acid, diisononyl ester; DNP, di-isononyl phthalate; WQS, weighted quantile sum; High−MWP, high molecular-weight phthalate; Ln, natural logarithm; Low−MWP, low molecular-weight phthalate; MW, Molecular Weight; OR, odds ratios; T2, second tertile; T3, third tertile. ^a^High-MWP is the molar sum of MCPP, MBzP, MHiBP, MEHP, MECPP, MEHHP, MEOHP, MCOP, MONP, MCNP, MHINCH, MCOCH, MEHHTP, and MECPTP. Low-MWP is the molar sum of MEP, MiBP, MHiBP, MBP, and MHBP. ΣDBP is the molar sum of MBP and MHBP. ΣDEHP is the molar sum of MECPP, MEHPP, MEOHP, and MEHP. ΣDEHTP is the molar sum of MEHHTP and MECPTP. ΣDiBP is the molar sum of MiBP and MHiBP. ΣDINCH is the molar sum of MHINCH and MCOCH. ΣDNP is the molar sum of MCOP and MONP. ^b^Tertile 1 is the reference category. ^c^The crude model did not account for covariates, while the adjusted model accounted for age, sex, race/ethnicity, poverty income ratio, marital status, education level, body mass index, physical activity, smoking and drinking status, and creatinine.

Figure 2 depicted the association between the sums of phthalate metabolites and gallstone occurrence. Following adjustments for confounding factors, logistic regression indicated a 31% increase in gallstone risk per unit rise in ΣDEHP and an 87% elevation for ΣDINCH. WQS regression revealed a 91% escalation in gallstone risk per interquartile range increase for High-MWP, 87% for ΣDINCH, and 57% for ΣDEHTP mixtures. The major contributors in High-MWP were MCOCH, MECPP, and MBzP, while in ΣDINCH, it was MCOCH, and in ΣDEHTP, it was MECPTP (Supplementary Figure S2). No significant associations were observed for Low-MWP, DBP, DiBP, and DNP with gallstones.

**Figure 3:**
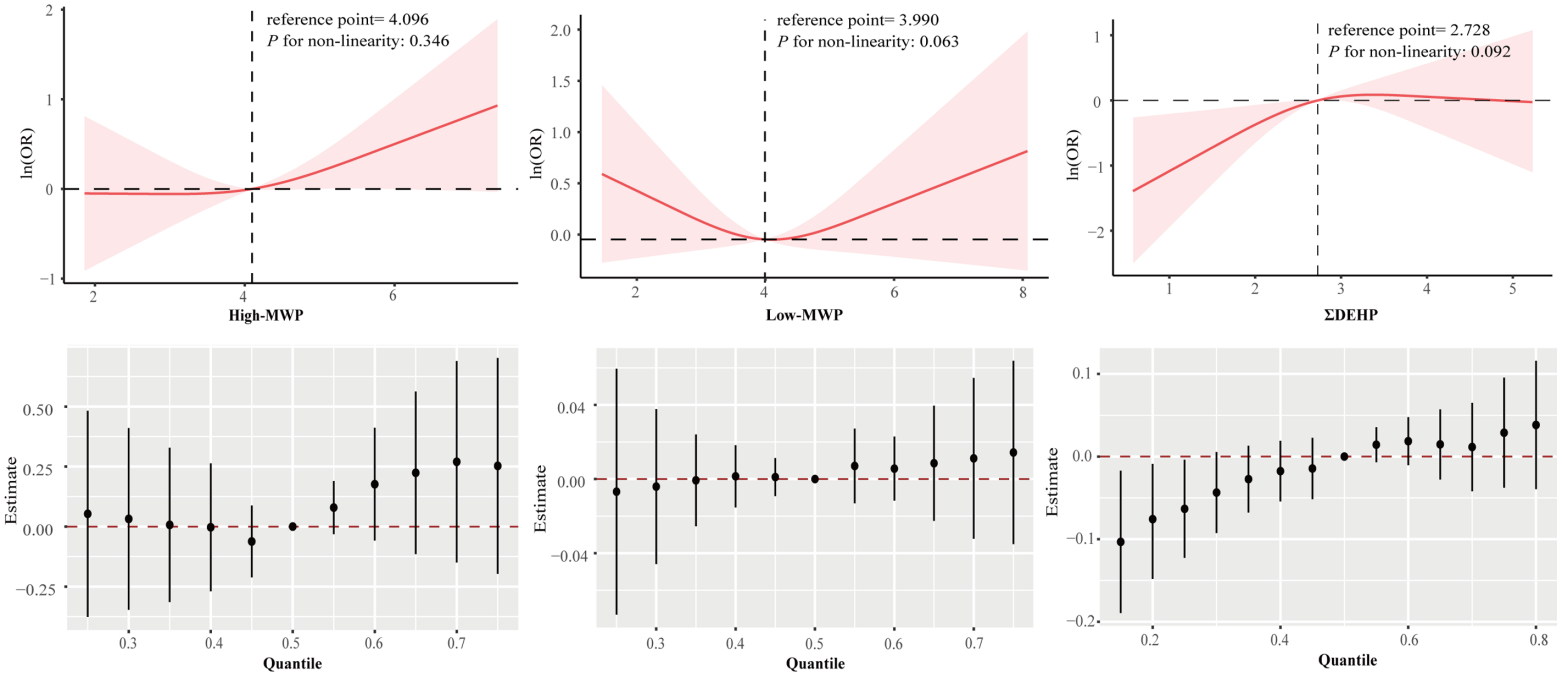
Association between sums phthalate metabolite and gallstone estimated by RCS and BKMR. Models were adjusted for age, sex, race/ethnicity, poverty income ratio, marital status, education level, body mass index, physical activity, smoking and drinking status, and creatinine.

RCS regression indicated a significant dose–response association with gallstones for the individual phthalate metabolites, such as MECPP, MEOHP, MHINCH, MCOCH, and MEHHTP, as well as for the sum phthalate metabolites, such as ΣDEHP and ΣDINCH (Supplementary Figures S3, S4; Figure 3). In BKMR analysis, the overall effect of mixtures was associated with a lower risk of gallstone when all DEHP metabolites were below the 25th percentile compared to their median (Figure 3), with the PIP for both MECPP and MEHHP exceeding the 0.5 threshold.

### Mediation and subgroup analysis

**Figure 4:**
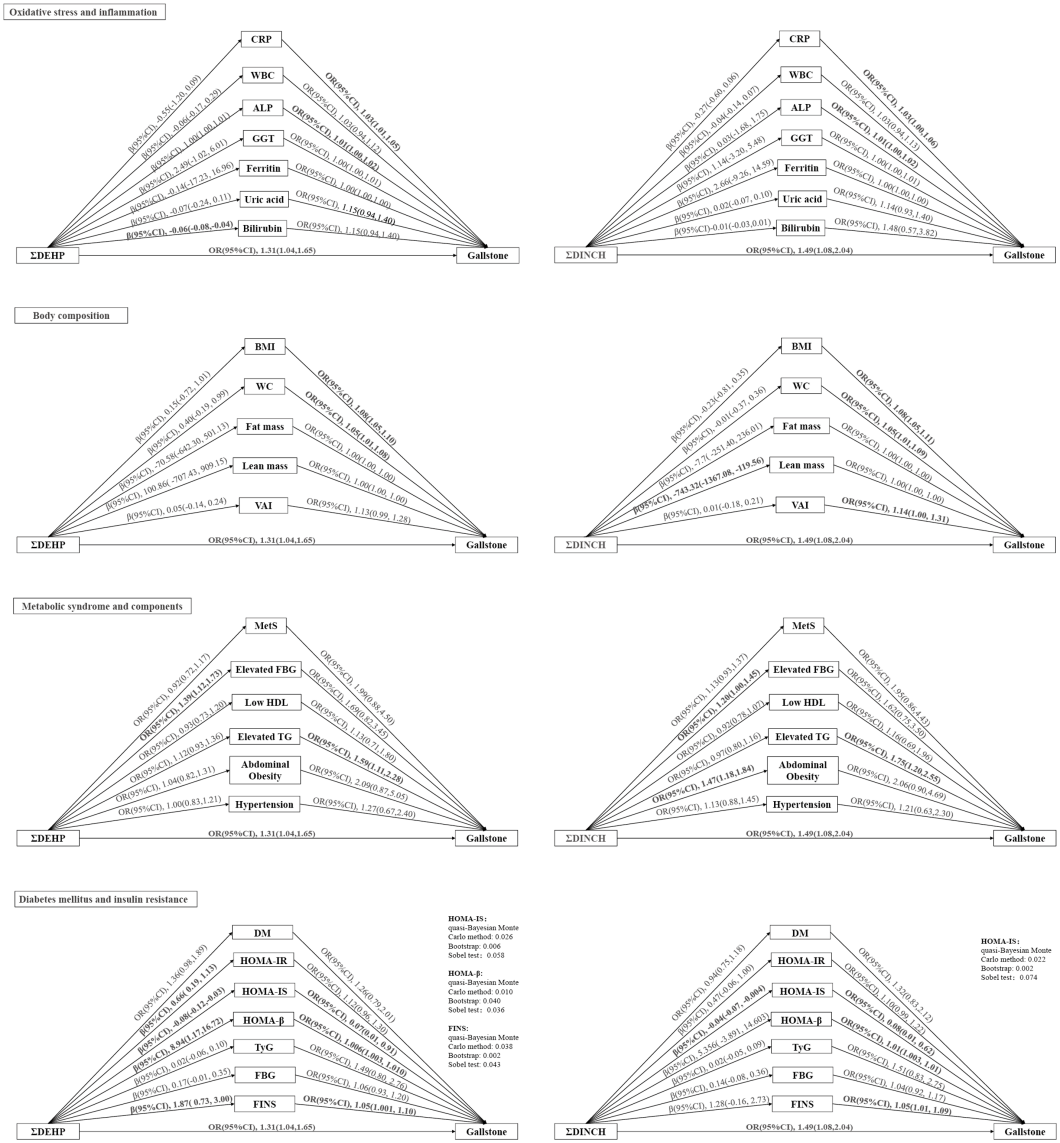
Mediation analysis of oxidative stress, inflammation, body composition, metabolic syndrome, diabetes mellitus, and insulin resistance in the association between sums phthalate metabolite and gallstone. Bold lines and fonts are used to indicate statistical significance (*p* < 0.05). Models were adjusted for age, sex, race/ethnicity, poverty income ratio, marital status, education level, body mass index (except when it was used as a mediator), physical activity, smoking and drinking status, and creatinine. ALP, alkaline phosphatase; BMI, body mass index; CI, confidence interval; CRP, C-reaction protein; DEHP, di(2-ethylhexyl) phthalate; DM, diabetes mellitus; DINCH, 1,2-Cyclohexane dicarboxylic acid, diisononyl ester; FBG, fasting blood glucose; FINS, fasting serum insulin; GGT, gamma glutamyl transferase; HDL, High-Density Lipoprotein; HOMA-IR, homeostatic model assessment of insulin resistance; HOMA-IS, homeostasis model assessment of insulin sensitivity; HOMA-β, homeostasis model assessment of β-cell function; MetS, metabolic syndrome; OR, odds ratios; TG, Triglyceride; TyG, triglyceride glucose index; VAI, visceral adiposity index; WBC, white blood cell; WC, waist circumference.

Given the significance of ΣDEHP and ΣDINCH in the adjusted multivariate logistic regression and RCS, our study has centered on exploring their potential mediating roles in relation to gallstones. Our study reveals that ΣDEHP is linked to higher levels of HOMA-IS, HOMA-β, and FINS. Notably, lower HOMA-IS and higher HOMA-β and FINS levels are significantly associated with a higher risk of gallstones (Figure 4). Multiple testing methods consistently highlight the significant mediating effects of these three indicators, suggesting that ΣDEHP may elevate FINS levels by reducing HOMA-IS and increasing HOMA-β, ultimately contributing to the occurrence of gallstones. Furthermore, we observed a significant mediating effect of HOMA-IS in the association between ΣDINCH and gallstones (Figure 4). However, no notable mediating effects were observed for indicators related to oxidative stress, inflammation, body composition, or metabolic syndrome.

No significant interactions were observed, indicating a consistent relationship between phthalate metabolites and gallstones across these subgroups (Supplementary Tables S2, S3).

## Discussion

In this nationally representative cross-sectional study, we found a significant association between phthalate and its metabolites (especially high MW metabolites, e.g., DEHP, DINCH) and gallstone. Furthermore, mediation analyses indicated that phthalate metabolites may play a role in the development of gallstones by influencing HOMA-IS, HOMA-β, and FINS. Subgroup analyses did not reveal significant interaction.

Given the widespread utilization of phthalates, prior research has extensively delved into examining the relationship between phthalates and various health outcomes, including serum insulin, type 2 diabetes mellitus, overweight, obesity, and skeletal abnormalities (16). Notably, specific phthalates (MMP, MiBP, and MEP) have demonstrated a robust correlation with an elevated prevalence of diabetes mellitus (43). This association may potentially arise from the impact of phthalic acid on serum insulin. Research conducted by Dales et al. has provided evidence supporting a strong association between phthalate exposure and increased serum insulin levels (44). Wei et al. further elucidates the role of oxidative stress in this process (45). Gaston et al.’s population-based study reinforces similar conclusions (46), underscoring the need for a more comprehensive exploration of the impact of phthalic acid on serum insulin and insulin sensitivity. However, it is important to note that this particular research area remains inadequately addressed.

Previous studies have examined diverse aspects of gallstones, exploring their connections with cardiovascular disease, metabolic syndrome, high-calorie intake, and alterations in serum insulin levels (47–49). Noteworthy is Chen’s investigation into gallstones and metabolic syndrome, which extensively explores the association between various indicators leading to metabolic syndrome and gallstones, with blood glucose and lipids identified as significant influencing factors (50). Moga’s study also observed an association between hyperinsulinemia and gallstones (51). Additionally, a study by Tsai et al. identifies high-calorie intake as a significant risk factor for gallstone formation, with an observed association of gallstones with insulin sensitivity and hyperinsulinemia in their macronutrient intake investigation (49), aligning with our findings.

The pathogenesis of phthalates and gallstones remains unclear, yet multiple potential mechanisms are hypothesized. Prior research indicates that exposure to phthalates diminishes insulin sensitivity. For instance, DEHP activates PPARs, diminishing insulin sensitivity while stimulating insulin secretion, consequently inducing hyperinsulinemia (20). Insulin triggers the activation of pivotal factors in cholesterol synthesis, including 3-hydroxy-3-methylglutaryl coenzyme A reductase (HMG-CoA), along with genes such as ATP-binding cassette transporters G5 and G8 (ABCG5/G8) responsible for cholesterol secretion, and the farnesoid X receptor (FXR) acting as a bile acid sensor (52). These interactions contribute to cholesterol secretion, thereby fostering gallstone formation. Moreover, reduced insulin sensitivity precipitates the catabolism of glycogen and lipids, elevating free fatty acids and increasing the influx of very low-density lipoprotein (VLDL) into the liver (53). This process enhances hepatic cholesterol secretion into bile, culminating in gallbladder bile supersaturation.

Previous research has established a positive association between urinary phthalates and inflammatory markers such as C-reactive protein (CRP), interleukin- 6 (IL-6), and tumor necrosis factor α (TNF-α) (54, 55). A study detected a noteworthy correlation between gallstones and systemic markers of inflammation, notably white blood cell counts and CRP. This finding implies that inflammation might serve as a potential causative mechanism in gallstone formation (56). Moreover, animal studies indicate that chronic exposure to phthalates like DEHP can disrupt the gut microbial balance in mice (57, 58). The dysbiosis of gut microbiota in patients with gallstones was also considered to play a significant role in the pathogenesis of gallstone disease (52). Reviews highlight how exposure to these compounds can disrupt lipid metabolism, potentially contributing to obesity (59). However, the meta-analysis showed that most of the results in the studies on the association between phthalates and obesity measures did not reach statistical significance, and the inconsistencies found between the studies did not allow for clear conclusions to be drawn (60).

Based on prior research and our study findings, we suggest that insulin and its metabolism serve as a significant mediator in the relationship between phthalate metabolites and the occurrence of gallstones, as opposed to oxidative stress, inflammation, body composition, and metabolic syndrome. However, given the nature of our study design and sample size, this conclusion should be interpreted with caution, and further validation is warranted through prospective research in the future.

To our knowledge, this is the first study examining the association between phthalate exposure and gallstone. The NHANES study participants constituted a representative sample from the United States, adhering to a well-designed study protocol with rigorous quality control measures, ensuring the reliability of our conclusions. However, the present study has several limitations. Firstly, its cross-sectional nature prevents the establishment of a causal relationship between gallstones and urinary phthalate levels. Secondly, the diagnosis of gallstones relied on a questionnaire, introducing the possibility of recall bias. Thirdly, clinical variables such as medication history and specific stone composition were unavailable in the database. Fourth, urinary phthalate levels were measured only once, lacking the ability to capture potential variations over time, which may hinder the accurate representation of long-term phthalate exposure and its association with outcomes. Additionally, when mediation analyses, the sample size was halved, potentially impacting the robustness of the findings. Despite efforts to adjust for numerous potential confounders, residual confounding (e.g., medical conditions, diet, occupation, drug use, other environmental chemicals), and unanticipated factors (e.g., genetic influences) could not be eliminated. Despite these limitations, the study successfully demonstrated an association between gallstones and urinary phthalates, emphasizing the need for future multicenter prospective cohort studies to delve deeper into this relationship.

## Conclusion

In this study, we demonstrated for a nationally representative sample of the population that exposure to some phthalate metabolites was associated with gallstones, potentially mediated by hyperinsulinemia. Future comprehensive research and interventions focusing on reducing phthalate metabolite exposure and managing insulin levels are expected to be pivotal in addressing and lowering the prevalence of gallstones.

## Data availability statement

The datasets presented in this study can be found in online repositories. The names of the repository/repositories and accession number(s) can be found at: https://www.cdc.gov/nchs/nhanes/index.htm.

## Ethics statement

The studies involving humans were approved by NCHS Research Ethics Review Committee. The studies were conducted in accordance with the local legislation and institutional requirements. The participants provided their written informed consent to participate in this study.

## Author contributions

HT: Conceptualization, Data curation, Formal analysis, Investigation, Methodology, Software, Visualization, Writing – original draft, Writing – review & editing. XZ: Data curation, Formal analysis, Investigation, Software, Supervision, Validation, Writing – original draft, Writing – review & editing. JH: Methodology, Software, Writing – original draft, Writing – review & editing. NL: Data curation, Formal analysis, Writing – original draft, Writing – review & editing. HC: Visualization, Writing – original draft. QY: Writing – original draft. HL: Writing – original draft. HH: Conceptualization, Data curation, Formal analysis, Investigation, Project administration, Supervision, Writing – review & editing.

## Glossary

**Table:** 

ABCG5/G8	ATP-binding cassette transporters G5 and G8
BMI	Body mass index
BKMR	Bayesian kernel machine regression
CRP	C-reactive protein
FBG	Fasting blood glucose
FINS	Fasting serum insulin
FXR	Farnesoid X receptor
High-MWP	High MW phthalate
HMG-CoA	3-hydroxy-3-methylglutaryl coenzyme A reductase
HOMA	Homeostasis model assessment
HOMA-β	Homeostasis model assessment β-cell function
HOMA-IR	Homeostasis model assessment insulin resistance
HOMA-IS	Homeostasis model assessment insulin sensitivity
HPLC-ESI-MS/MS	High-performance liquid chromatography-electrospray ionization-tandem mass spectrometry
IL-6	Interleukin- 6
LOD	Limit of detection
Low-MWP	Low MW phthalate
MBP	Mono-n-butyl phthalate
MBzP	Monobenzyl phthalate
MCNP	Monocarboxy-isononyl phthalate
MCOCH	Cyclohexane-1,2-dicarboxylic acid mono(carboxyoctyl) ester
MCOP	Monocarboxyisooctyl phthalate
MCPP	Mono (3-carboxypropyl) phthalate
MECPP	Mono(2-ethyl-5-carboxypentyl) phthalate
MECPTP	Mono(2-ethyl-5-carboxypentyl) terephthalate
MEHHP	Mono(2-ethyl-5-hydroxyhexyl) phthalate
MEHHTP	Mono(2-ethyl-5-hydroxyhexyl) terephthalate
MEHP	Mono(2-ethylhexyl) phthalate
MEOHP	Mono(2-ethyl-5-oxohexyl) phthalate
MEP	Mono-ethyl phthalate
MHBP	Mono-3-hydroxybutyl phthalate
MHiBP	Mono-2-hydroxy-iso-butyl phthalate
MHINCH	Cyclohexane-1,2-dicarboxylic acid mono(hydroxy-isononyl) ester
MiBP	Mono-isobutyl phthalate
MNP	Mono-isononyl phthalate
MONP	Mono-oxo-isononyl phthalate
MW	Molecular weight
NCHS	National Center for Health Statistics
NHANES	National Health and Nutrition Examination Survey
PA	Physical activity
PIPs	Posterior incorporation probabilities
PIR	Poverty income ratio
PPARs	Peroxisome proliferator-activated receptors
RCS	Restricted cubic spline
SE	Standard error
SPE	Solid phase extraction
TNF-α	Tumor necrosis factor α
VLDL	Very low-density lipoprotein
WQS	Weighted quantile sum
ΣDBP	Di-n-butyl phthalate
ΣDEHP	Di(2-ethylhexyl) phthalate
ΣDEHTP	Di(2-ethylhexyl) terephthalate
ΣDiBP	Di-isobutyl phthalate
ΣDINCH	1,2-Cyclohexane dicarboxylic acid, diisononyl ester
ΣDNP	Di-isononyl phthalate

## References

[1] Lammert F Gurusamy K Ko CW Miquel J-F Méndez-Sánchez N Portincasa P. Gallstones. Nat Rev Dis Primers. (2016) 2:16024. doi: 10.1038/nrdp.2016.24 27121416

[2] Gurusamy KS Davidson BR. Gallstones. BMJ. (2014) 348:g2669. doi: 10.1136/bmj.g2669 24755732

[3] Gutt C Schläfer S Lammert F. The treatment of gallstone disease. Dtsch Arztebl Int. (2020) 117:148–58. doi: 10.3238/arztebl.2020.0148 32234195 PMC7132079

[4] Zhu Q Sun X Ji X Zhu L Xu J Wang C. The association between gallstones and metabolic syndrome in urban Han Chinese: a longitudinal cohort study. Sci Rep. (2016) 6:29937. doi: 10.1038/srep29937, PMID: 27443986 PMC4957232

[5] Stinton LM Shaffer EA. Epidemiology of gallbladder disease: cholelithiasis and cancer. Gut Liver. (2012) 6:172–87. doi: 10.5009/gnl.2012.6.2.172, PMID: 22570746 PMC3343155

[6] Af P Sd C Cc M Jl L Es D Jl W. Burden and cost of gastrointestinal, liver, and pancreatic diseases in the United States: update 2018. Gastroenterology. (2019) 156:254–272.e11. doi: 10.1053/j.gastro.2018.08.063, PMID: 30315778 PMC6689327

[7] Song ST Shi J Wang XH Guo YB Hu PF Zhu F. Prevalence and risk factors for gallstone disease: a population-based cross-sectional study. J Dig Dis. (2020) 21:237–45. doi: 10.1111/1751-2980.12857, PMID: 32166900

[8] Lee M-H Gao Y-T Huang Y-H McGee EE Lam T Wang B. A metallomic approach to assess associations of serum metal levels with gallstones and gallbladder Cancer. Hepatology. (2020) 71:917–28. doi: 10.1002/hep.30861, PMID: 31318976 PMC6980252

[9] Zota AR Calafat AM Woodruff TJ. Temporal trends in phthalate exposures: findings from the National Health and nutrition examination survey, 2001-2010. Environ Health Perspect. (2014) 122:235–41. doi: 10.1289/ehp.1306681, PMID: 24425099 PMC3948032

[10] Meeker JD Sathyanarayana S Swan SH. Phthalates and other additives in plastics: human exposure and associated health outcomes. Philos Trans R Soc Lond Ser B Biol Sci. (2009) 364:2097–113. doi: 10.1098/rstb.2008.0268, PMID: 19528058 PMC2873014

[11] Net S Sempéré R Delmont A Paluselli A Ouddane B. Occurrence, fate, behavior and ecotoxicological state of phthalates in different environmental matrices. Environ Sci Technol. (2015) 49:4019–35. doi: 10.1021/es505233b, PMID: 25730609

[12] Bornehag C-G Sundell J Weschler CJ Sigsgaard T Lundgren B Hasselgren M. The association between asthma and allergic symptoms in children and phthalates in house dust: a nested case-control study. Environ Health Perspect. (2004) 112:1393–7. doi: 10.1289/ehp.7187, PMID: 15471731 PMC1247566

[13] Yang Y Ju L Fan J Cai S Sun L Li Y. Association of urinary phthalate metabolites with sarcopenia in US adults: NHANES 1999-2006. Environ Sci Pollut Res Int. (2022) 29:7573–82. doi: 10.1007/s11356-021-16202-5 34480309

[14] Guo Y Alomirah H Cho H-S Minh TB Mohd MA Nakata H. Occurrence of phthalate metabolites in human urine from several Asian countries. Environ Sci Technol. (2011) 45:3138–44. doi: 10.1021/es103879m, PMID: 21395215

[15] Kehagias D Kostopoulou E Ravazoula P Panagopoulos K. Thyroid angiosarcoma (TAS) – a rare diagnosis not to be missed. Clin Case Rep. (2021) 9:173–6. doi: 10.1002/ccr3.3492, PMID: 33489155 PMC7813081

[16] Benjamin S Masai E Kamimura N Takahashi K Anderson RC Faisal PA. Phthalates impact human health: epidemiological evidences and plausible mechanism of action. J Hazard Mater. (2017) 340:360–83. doi: 10.1016/j.jhazmat.2017.06.036, PMID: 28800814

[17] Stojanoska MM Milosevic N Milic N Abenavoli L. The influence of phthalates and bisphenol a on the obesity development and glucose metabolism disorders. Endocrine. (2017) 55:666–81. doi: 10.1007/s12020-016-1158-4, PMID: 27822670

[18] Bełtowski J Wójcicka G Jamroz-Wiśniewska A. Hydrogen sulfide in the regulation of insulin secretion and insulin sensitivity: implications for the pathogenesis and treatment of diabetes mellitus. Biochem Pharmacol. (2018) 149:60–76. doi: 10.1016/j.bcp.2018.01.004, PMID: 29307653

[19] Yang R Zheng J Qin J Liu S Liu X Gu Y. Dibutyl phthalate affects insulin synthesis and secretion by regulating the mitochondrial apoptotic pathway and oxidative stress in rat insulinoma cells. Ecotoxicol Environ Saf. (2023) 249:114396. doi: 10.1016/j.ecoenv.2022.114396, PMID: 36508788

[20] Latini G Marcovecchio ML Del Vecchio A Gallo F Bertino E Chiarelli F. Influence of environment on insulin sensitivity. Environ Int. (2009) 35:987–93. doi: 10.1016/j.envint.2009.03.008 19395033

[21] Ruhl CE Everhart JE. Association of diabetes, serum insulin, and C-peptide with gallbladder disease. Hepatology. (2000) 31:299–303. doi: 10.1002/hep.510310206, PMID: 10655249

[22] Kim JM Lee HL Moon W Koh DH Lee OY Yoon BC. Association between insulin, insulin resistance, and gallstone disease in Korean general population. Korean J Gastroenterol. (2007) 50:183–7. doi: 10.14309/00000434-200609001-00181 PMID: 17885284

[23] CDC. (2017–2018) NHANES Questionnaires, Datasets, and Related Documentation. Available at: https://wwwn.cdc.gov/nchs/nhanes/continuousnhanes/ (Accessed December 20, 2023)

[24] Wang J Sun Y-X Xiang S Yang C Li X-J Zhang M-Q. The association between blood heavy metals and gallstones: a cross-sectional study. Sci Total Environ. (2023) 904:166735. doi: 10.1016/j.scitotenv.2023.166735, PMID: 37659556

[25] Kato K Silva MJ Needham LL Calafat AM. Determination of 16 phthalate metabolites in urine using automated sample preparation and on-line preconcentration/high-performance liquid chromatography/tandem mass spectrometry. Anal Chem. (2005) 77:2985–91. doi: 10.1021/ac0481248, PMID: 15859620

[26] Li M-C Lin C-Y Guo YL. Urinary concentrations of phthalates in relation to circulating fatty acid profile in National Health and nutrition examination survey, 2003-2004 and 2011-2012. Environ Pollut. (2020) 265:114714. doi: 10.1016/j.envpol.2020.114714, PMID: 32540591

[27] Wolff MS Teitelbaum SL Pinney SM Windham G Liao L Biro F. Investigation of relationships between urinary biomarkers of phytoestrogens, phthalates, and phenols and pubertal stages in girls. Environ Health Perspect. (2010) 118:1039–46. doi: 10.1289/ehp.0901690, PMID: 20308033 PMC2920905

[28] Wang C-J Yang H-W Li M-C. Association between phthalate exposure and the risk of depressive symptoms in the adult population of the United States. Chemosphere. (2023) 334:139031. doi: 10.1016/j.chemosphere.2023.139031, PMID: 37244561

[29] Yun L Vanderloo LM Berry TR Latimer-Cheung AE O’Reilly N Rhodes RE. Political orientation and public attributions for the causes and solutions of physical inactivity in Canada: implications for policy support. Front Public Health. (2019) 7:153. doi: 10.3389/fpubh.2019.00153, PMID: 31316958 PMC6611409

[30] Peng H Yeh F Lin J Best LG Cole SA Lee ET. Plasminogen activator inhibitor-1 is associated with leukocyte telomere length in American Indians: findings from the strong heart family study. J Thromb Haemost. (2017) 15:1078–85. doi: 10.1111/jth.13689, PMID: 28378522 PMC5500969

[31] Rattan P Penrice DD Ahn JC Ferrer A Patnaik M Shah VH. Inverse Association of Telomere Length with Liver Disease and Mortality in the US population. Hepatol Commun. (2022) 6:399–410. doi: 10.1002/hep4.1803, PMID: 34558851 PMC8793996

[32] Carrico C Gennings C Wheeler DC Factor-Litvak P. Characterization of weighted quantile sum regression for highly correlated data in a risk analysis setting. J Agric Biol Environ Stat. (2015) 20:100–20. doi: 10.1007/s13253-014-0180-3, PMID: 30505142 PMC6261506

[33] Bobb JF Valeri L Claus Henn B Christiani DC Wright RO Mazumdar M. Bayesian kernel machine regression for estimating the health effects of multi-pollutant mixtures. Biostatistics. (2015) 16:493–508. doi: 10.1093/biostatistics/kxu058, PMID: 25532525 PMC5963470

[34] Liu W Wang J Wang M Hou H Ding X Ma L. Oxidative stress factors mediate the association between Life’s essential 8 and accelerated phenotypic aging: NHANES 2005-2018. J Gerontol A Biol Sci Med Sci. (2023) 79:glad240. doi: 10.1093/gerona/glad240, PMID: 37813096

[35] Ferguson KK Loch-Caruso R Meeker JD. Exploration of oxidative stress and inflammatory markers in relation to urinary phthalate metabolites: NHANES 1999-2006. Environ Sci Technol. (2012) 46:477–85. doi: 10.1021/es202340b, PMID: 22085025 PMC3258337

[36] Grundy SM Cleeman JI Daniels SR Donato KA Eckel RH Franklin BA. Diagnosis and management of the metabolic syndrome: an American Heart Association/National Heart, Lung, and Blood Institute scientific statement. Circulation. (2005) 112:2735–52. doi: 10.1161/CIRCULATIONAHA.105.169404 16157765

[37] Amato MC Giordano C. Visceral adiposity index: an indicator of adipose tissue dysfunction. Int J Endocrinol. (2014) 2014:730827. doi: 10.1155/2014/730827, PMID: 24829577 PMC4009335

[38] Baker JF Weber DR Neogi T George MD Long J Helget LN. Associations between low serum urate, body composition, and mortality. Arthritis Rheumatol. (2023) 75:133–40. doi: 10.1002/art.42301, PMID: 35974440 PMC10600587

[39] Guerrero-Romero F Simental-Mendía LE González-Ortiz M Martínez-Abundis E Ramos-Zavala MG Hernández-González SO. The product of triglycerides and glucose, a simple measure of insulin sensitivity. Comparison with the euglycemic-hyperinsulinemic clamp. J Clin Endocrinol Metab. (2010) 95:3347–51. doi: 10.1210/jc.2010-0288, PMID: 20484475

[40] Matthews DR Hosker JP Rudenski AS Naylor BA Treacher DF Turner RC. Homeostasis model assessment: insulin resistance and beta-cell function from fasting plasma glucose and insulin concentrations in man. Diabetologia. (1985) 28:412–9. doi: 10.1007/BF00280883, PMID: 3899825

[41] Imai K Keele L Tingley D. A general approach to causal mediation analysis. Psychol Methods. (2010) 15:309–34. doi: 10.1037/a0020761 20954780

[42] Sobel ME. Asymptotic confidence intervals for indirect effects in structural equation models. Sociol Methodol. (1982) 13:290. doi: 10.2307/270723

[43] Lind PM Zethelius B Lind L. Circulating levels of phthalate metabolites are associated with prevalent diabetes in the elderly. Diabetes Care. (2012) 35:1519–24. doi: 10.2337/dc11-2396, PMID: 22498808 PMC3379584

[44] Dales RE Kauri LM Cakmak S. The associations between phthalate exposure and insulin resistance, β-cell function and blood glucose control in a population-based sample. Sci Total Environ. (2018) 612:1287–92. doi: 10.1016/j.scitotenv.2017.09.009, PMID: 28898934

[45] Wei J Hao Q Chen C Li J Han X Lei Z. Epigenetic repression of miR-17 contributed to di(2-ethylhexyl) phthalate-triggered insulin resistance by targeting Keap1-Nrf2/miR-200a axis in skeletal muscle. Theranostics. (2020) 10:9230–48. doi: 10.7150/thno.45253, PMID: 32802189 PMC7415800

[46] Gaston SA Tulve NS. Urinary phthalate metabolites and metabolic syndrome in U.S. adolescents: cross-sectional results from the National Health and nutrition examination survey (2003-2014) data. Int J Hyg Environ Health. (2019) 222:195–204. doi: 10.1016/j.ijheh.2018.09.005, PMID: 30297147 PMC11780690

[47] Méndez-Sánchez N Bahena-Aponte J Chávez-Tapia NC Motola-Kuba D Sánchez-Lara K Ponciano-Radríguez G. Strong association between gallstones and cardiovascular disease. Am J Gastroenterol. (2005) 100:827–30. doi: 10.1111/j.1572-0241.2005.41214.x, PMID: 15784027

[48] Zhu Q Xing Y Fu Y Chen X Guan L Liao F. Causal association between metabolic syndrome and cholelithiasis: a Mendelian randomization study. Front Endocrinol (Lausanne). (2023) 14:1180903. doi: 10.3389/fendo.2023.1180903, PMID: 37361524 PMC10288183

[49] Tsai C-J Leitzmann MF Willett WC Giovannucci EL. Macronutrients and insulin resistance in cholesterol gallstone disease. Am J Gastroenterol. (2008) 103:2932–9. doi: 10.1111/j.1572-0241.2008.02189.x, PMID: 18853969

[50] Chen L-Y Qiao Q-H Zhang S-C Chen Y-H Chao G-Q Fang L-Z. Metabolic syndrome and gallstone disease. World J Gastroenterol. (2012) 18:4215–20. doi: 10.3748/wjg.v18.i31.4215, PMID: 22919256 PMC3422804

[51] Moga MM. Alternative treatment of gallbladder disease. Med Hypotheses. (2003) 60:143–7. doi: 10.1016/s0306-9877(02)00351-1 12450782

[52] Di Ciaula A Wang DQ-H Portincasa P. An update on the pathogenesis of cholesterol gallstone disease. Curr Opin Gastroenterol. (2018) 34:71–80. doi: 10.1097/MOG.0000000000000423, PMID: 29283909 PMC8118137

[53] Taskinen M-R. Type 2 diabetes as a lipid disorder. Curr Mol Med. (2005) 5:297–308. doi: 10.2174/1566524053766086, PMID: 15892649

[54] Bai PY Wittert G Taylor AW Martin SA Milne RW Jenkins AJ. The association between total phthalate concentration and non-communicable diseases and chronic inflammation in south Australian urban dwelling men. Environ Res. (2017) 158:366–72. doi: 10.1016/j.envres.2017.06.021, PMID: 28686951

[55] Ferguson KK Loch-Caruso R Meeker JD. Urinary phthalate metabolites in relation to biomarkers of inflammation and oxidative stress: NHANES 1999-2006. Environ Res. (2011) 111:718–26. doi: 10.1016/j.envres.2011.02.002, PMID: 21349512 PMC3110976

[56] Shabanzadeh DM Skaaby T Sørensen LT Eugen-Olsen J Jørgensen T. Metabolic biomarkers and gallstone disease – a population-based study. Scand J Gastroenterol. (2017) 52:1270–7. doi: 10.1080/00365521.2017.1365166 28799434

[57] Su H Yuan P Lei H Zhang L Deng D Zhang L. Long-term chronic exposure to di-(2-ethylhexyl)-phthalate induces obesity via disruption of host lipid metabolism and gut microbiota in mice. Chemosphere. (2022) 287:132414. doi: 10.1016/j.chemosphere.2021.132414, PMID: 34600010

[58] Almamoun R Pierozan P Manoharan L Karlsson O. Altered gut microbiota community structure and correlated immune system changes in dibutyl phthalate exposed mice. Ecotoxicol Environ Saf. (2023) 262:115321. doi: 10.1016/j.ecoenv.2023.115321, PMID: 37549549

[59] Chang W-H Herianto S Lee C-C Hung H Chen H-L. The effects of phthalate ester exposure on human health: a review. Sci Total Environ. (2021) 786:147371. doi: 10.1016/j.scitotenv.2021.147371, PMID: 33965815

[60] Ribeiro C Mendes V Peleteiro B Delgado I Araújo J Aggerbeck M. Association between the exposure to phthalates and adiposity: a meta-analysis in children and adults. Environ Res. (2019) 179:108780. doi: 10.1016/j.envres.2019.108780, PMID: 31610390

