# Supplementary material for: Phthalate and gallstones: the mediation of insulin
Source: Front Public Health. 2024 Jun 5;12:1401420. doi: 10.3389/fpubh.2024.1401420 (PMC11188473; doi:10.3389/fpubh.2024.1401420)
Supplement: Supplementary file 1 [file Data_Sheet_1.docx]

**Supplementary Materials Files**

To: **Phthalate and Gallstones: The Mediation of Insulin**

by Haoxian Tang, Xuan Zhang, Jingtao Huang, Nan Luo, Hongyu Chen, Qinglong Yang, Hanyuan Lin, Hao Hua.

**Supplementary Table**

Supplementary Table 1. Phthalates and phthalate alternatives and their metabolites used as biomarkers of exposure.

Supplementary Table 2. Association between sums phthalate metabolite (High-MWP, ΣDEHP, ΣDEHTP, and ΣDINCH) and gallstone among different subgroups.

Supplementary Table 3. Association between sums phthalate metabolite (Low-MWP, ΣDBP, ΣDiBP, and ΣDNP) and gallstone among different subgroups.

**Supplementary Figure**

Supplementary Figure 1. Flow Diagram of the Screening of Study Participants.

Supplementary Figure 2. WQS model regression index weights for gallstone.

Supplementary Figure 3. Association between individual phthalate metabolite and gallstone using restricted cubic splines regression.

Supplementary Figure 4. Association between sums phthalate metabolite and gallstone using restricted cubic splines regression.

Supplementary Figure 5. Posterior incorporation probabilities from the BKMR model.

**Supplementary Table 1. Phthalates and phthalate alternatives and their metabolites used as biomarkers of exposure.**

| **Parent chemical** | **Metabolite** | **NHANES Variable Name** | **Limits of detection (ng/mL)** | **Molecular Weight (g/mol)** |
| --- | --- | --- | --- | --- |
| Di-n-octyl phthalate (DOP)  Di-n-butyl phthalate (DBP)  Other high molecular weight phthalates | Mono (3-carboxypropyl) phthalate (MCPP) | urxmc1 | 0.4 | 252.2 |
| Di-ethyl phthalate (DEP) | Mono-ethyl phthalate (MEP) | urxmep | 1.2 | 194.2 |
| Di-isobutyl phthalate (DiBP) | Mono-isobutyl phthalate (MiBP)  Mono-2-hydroxy-iso-butyl phthalate (MHiBP) | urxmib  urxhibp | 0.8  0.4 | 222.2  237.2 |
| Di-n-butyl phthalate (DBP) | Mono-n-butyl phthalate (MBP)  Mono-3-hydroxybutyl phthalate (MHBP) | urxmbp  urxmhbp | 0.4  0.4 | 222.2  238.2 |
| Benzylbutyl phthalate (BzBP) | Monobenzyl phthalate (MBzP) | urxmzp | 0.3 | 256.3 |
| Di(2-ethylhexyl) phthalate (DEHP) | Mono(2-ethylhexyl) phthalate (MEHP)  Mono(2-ethyl-5-carboxypentyl) phthalate (MECPP)  Mono(2-ethyl-5-hydroxyhexyl) phthalate (MEHHP)  Mono(2-ethyl-5-oxohexyl) phthalate (MEOHP) | urxmhp  urxecp  urxmhh  urxmoh | 0.8  0.4  0.4  0.2 | 278.2  308.3  294.3  292.3 |
| Di-isononyl phthalate (DNP)* | *Mono-isononyl phthalate (MNP) ^#^  *Monocarboxyisooctyl phthalate (MCOP)  *Mono-oxo-isononyl phthalate (MONP) | urxmnp  urxcop  urxmonp | 0.9  0.3  0.4 | 222.2  322.4  306.4 |
| Di-isodecyl phthalate (DDP)* | *Monocarboxy-isononyl phthalate (MCNP) | urxcnp | 0.2 | 336.4 |
| 1,2-Cyclohexane dicarboxylic acid, diisononyl ester (DINCH)* | *Cyclohexane-1,2-dicarboxylic acid mono(hydroxy-isononyl) ester (MHINCH)  *Cyclohexane-1,2-dicarboxylic acid mono(carboxyoctyl) ester (MCOCH) | urxmhnc  urxmcoh | 0.4  0.5 | 314.4  328.4 |
| Di(2-ethylhexyl) terephthalate (DEHTP) | Mono(2-ethyl-5-hydroxyhexyl) terephthalate (MEHHTP)  Mono(2-ethyl-5-carboxypentyl) terephthalate (MECPTP) | urxmhht  urxecpt | 0.4  0.2 | 294.3  308.3 |

^*^isomeric mixtures. ^#.^ Most participants were below the lower limit of detection and were not included in the analysis.

**Supplementary Table 2. Association between sums phthalate metabolite (High-MWP, ΣDEHP, ΣDEHTP, and ΣDINCH) and gallstone among different subgroups.**

|  | **High-MWP** | | **ΣDEHP** | | **ΣDEHTP** | | **ΣDINCH** | |
| --- | --- | --- | --- | --- | --- | --- | --- | --- |
|  | **OR(95%CI)** | ***P* value** | **OR(95%CI)** | ***P* value** | **OR(95%CI)** | ***P* value** | **OR(95%CI)** | ***P* value** |
| **Age group** |  | 0.55^*^ |  | 0.40^*^ |  | 0.27^*^ |  | 0.75^*^ |
| <40 | 1.08(0.72-1.62) | 0.70 | 1.43(0.77-2.68) | 0.24 | 1.06(0.77-1.47) | 0.71 | 1.67(0.97-2.85) | 0.06 |
| 40-59 | 0.73(0.30-1.78) | 0.46 | 0.48(0.20-1.13) | 0.09 | 0.75(0.51-1.10) | 0.13 | 1.27(0.85-1.90) | 0.22 |
| ≥60 | 1.59(1.11-2.28) | 0.01 | 2.42(1.45-4.03) | 0.002 | 1.19(0.91-1.54) | 0.18 | 1.61(1.01-2.59) | 0.05 |
| **Sex** |  | 0.67^*^ |  | 0.11^*^ |  | 0.51^*^ |  | 0.13^*^ |
| Female | 1.04(0.66-1.65) | 0.85 | 1.57(1.20- 2.05) | 0.003 | 0.92(0.65- 1.28) | 0.58 | 1.15(0.78- 1.70) | 0.47 |
| Male | 1.51(0.88-2.57) | 0.12 | 0.95(0.54-1.68) | 0.85 | 1.26(0.91-1.74) | 0.15 | 2.06(1.33-3.17) | 0.003 |
| **Race/ethnicity** |  | 0.14^*^ |  | 0.05^*^ |  | 0.25^*^ |  | 0.28^*^ |
| Mexican American | 1.74(0.54-5.61) | 0.31 | 0.77(0.24-2.52) | 0.63 | 1.57(0.84-2.94) | 0.14 | 1.42(0.36-5.54) | 0.58 |
| Non-Hispanic Black | 1.05(0.64-1.71) | 0.84 | 1.26(0.84-1.89) | 0.24 | 1.05(0.76-1.45) | 0.75 | 1.61(0.96-2.72) | 0.07 |
| Non-Hispanic White | 1.22(0.82-1.82) | 0.31 | 1.45(0.93-2.26) | 0.10 | 1.00(0.74-1.35) | 0.98 | 1.49(1.07-2.09) | 0.02 |
| Other Hispanic | 0.88(0.66-1.16) | 0.33 | 1.33(0.73-2.41) | 0.32 | 0.91(0.66-1.25) | 0.54 | 1.33(0.69-2.56) | 0.36 |
| Other Race | 1.48(0.80-2.73) | 0.19 | 1.42(0.78-2.58) | 0.23 | 1.16(0.75-1.79) | 0.47 | 1.59(1.05-2.43) | 0.03 |
| **Body mass index** |  | 0.36^*^ |  | 0.52^*^ |  | 0.47^*^ |  | 0.09^*^ |
| <25 | 0.68(0.28-1.70) | 0.39 | 0.55(0.21-1.41) | 0.19 | 0.91(0.49-1.70) | 0.75 | 1.65(0.67-4.04) | 0.25 |
| 25-29 | 1.61(1.11-2.34) | 0.02 | 1.33(0.64-2.79) | 0.42 | 1.30(1.02-1.65) | 0.03 | 2.21(1.56-3.14) | <0.001 |
| ≥30 | 1.14(0.77-1.70) | 0.48 | 1.56(0.93-2.62) | 0.09 | 0.98(0.76-1.27) | 0.88 | 1.22(0.84-1.77) | 0.28 |

^*.^ *P* for interaction. Models were adjusted for age, sex, race/ethnicity, poverty income ratio, marital status, education level, body mass index, physical activity, smoking and drinking status, and creatinine. **Abbreviations:** CI, confidence interval; DEHP, di(2-ethylhexyl) phthalate; DiBP, di-isobutyl phthalate; DNP, di-isononyl phthalate; Low−MWP, low molecular-weight phthalate; OR, odds ratios.

**Supplementary Table 3. Association between sums phthalate metabolite (Low-MWP, ΣDBP, ΣDiBP, and ΣDNP) and gallstone among different subgroups.**

|  | **Low-MWP** | | **ΣDBP** | | **ΣDiBP** | | **ΣDNP** | |
| --- | --- | --- | --- | --- | --- | --- | --- | --- |
|  | **OR(95%CI)** | ***P* value** | **OR(95%CI)** | ***P* value** | **OR(95%CI)** | ***P* value** | **OR(95%CI)** | ***P* value** |
| **Age group** |  | 0.99^*^ |  | 0.97^*^ |  | 0.64^*^ |  | 0.59^*^ |
| <40 | 1.06(0.55-2.03) | 0.86 | 0.97(0.49-1.92) | 0.93 | 1.96(0.97-3.94) | 0.06 | 0.83(0.38-1.79) | 0.61 |
| 40-59 | 0.76(0.36-1.61) | 0.45 | 0.71(0.20-2.49) | 0.57 | 0.95(0.32-2.75) | 0.91 | 1.18(0.56-2.48) | 0.65 |
| ≥60 | 1.17(0.74-1.85) | 0.49 | 1.75(0.85-3.61) | 0.12 | 1.04(0.69-1.55) | 0.85 | 1.29(0.88-1.87) | 0.18 |
| **Sex** |  | 0.20^*^ |  | 0.23^*^ |  | 0.40^*^ |  | 0.78^*^ |
| Female | 1.06(0.76-1.47) | 0.71 | 1.39(0.78-2.45) | 0.24 | 1.18(0.90-1.54) | 0.22 | 1.17(0.93-1.47) | 0.17 |
| Male | 0.88(0.48-1.61) | 0.65 | 0.69(0.20-2.36) | 0.53 | 0.95(0.33-2.78) | 0.92 | 1.19(0.69-2.07) | 0.5 |
| **Race/ethnicity** |  | 0.11^*^ |  | 0.05^*^ |  | 0.11^*^ |  | 0.20^*^ |
| Mexican American | 1.51(0.97-2.36) | 0.07 | 2.21(1.10-4.45) | 0.03 | 1.52(0.85-2.71) | 0.14 | 0.73(0.30-1.74) | 0.43 |
| Non-Hispanic Black | 1.37(0.95-1.98) | 0.09 | 1.41(0.81-2.44) | 0.2 | 1.49(0.81-2.76) | 0.18 | 0.73(0.44-1.21) | 0.20 |
| Non-Hispanic White | 0.87(0.51-1.49) | 0.6 | 1.10(0.60-2.00) | 0.74 | 1.01(0.61-1.65) | 0.98 | 1.33(0.92-1.94) | 0.12 |
| Other Hispanic | 3.21(1.10-9.39) | 0.04 | 1.98(0.86-4.53) | 0.1 | 2.70(0.71-10.32) | 0.13 | 0.87(0.45-1.69) | 0.66 |
| Other Race | 1.11(0.72-1.72) | 0.61 | 1.20(0.57-2.49) | 0.61 | 0.99(0.41-2.39) | 0.98 | 1.76(0.77-4.03) | 0.17 |
| **Body mass index** |  | 0.57^*^ |  | 0.13^*^ |  | 0.08^*^ |  | 0.28^*^ |
| <25 | 0.50(0.25-1.02) | 0.05 | 0.31(0.15-0.65) | 0.004 | 0.09(0.02-0.36) | 0.002 | 0.91(0.40-2.03) | 0.80 |
| 25-29 | 0.74(0.39-1.39) | 0.32 | 1.17(0.42-3.28) | 0.75 | 0.94(0.47-1.87) | 0.85 | 1.22(0.77-1.92) | 0.38 |
| ≥30 | 1.15(0.80-1.65) | 0.43 | 1.38(0.93-2.07) | 0.11 | 1.45(1.01-2.08) | 0.04 | 1.13(0.78-1.63) | 0.50 |

^*.^ *P* for interaction. Models were adjusted for age, sex, race/ethnicity, poverty income ratio, marital status, education level, body mass index, physical activity, smoking and drinking status, and creatinine. **Abbreviations:** CI, confidence interval; DBP, di-n-butyl phthalate; DiBP, di-isobutyl phthalate; DNP, di-isononyl phthalate; High−MWP, high molecular-weight phthalate; OR, odds ratios.

**Supplementary Figure 1. Flow Diagram of the Screening of Study Participants.**


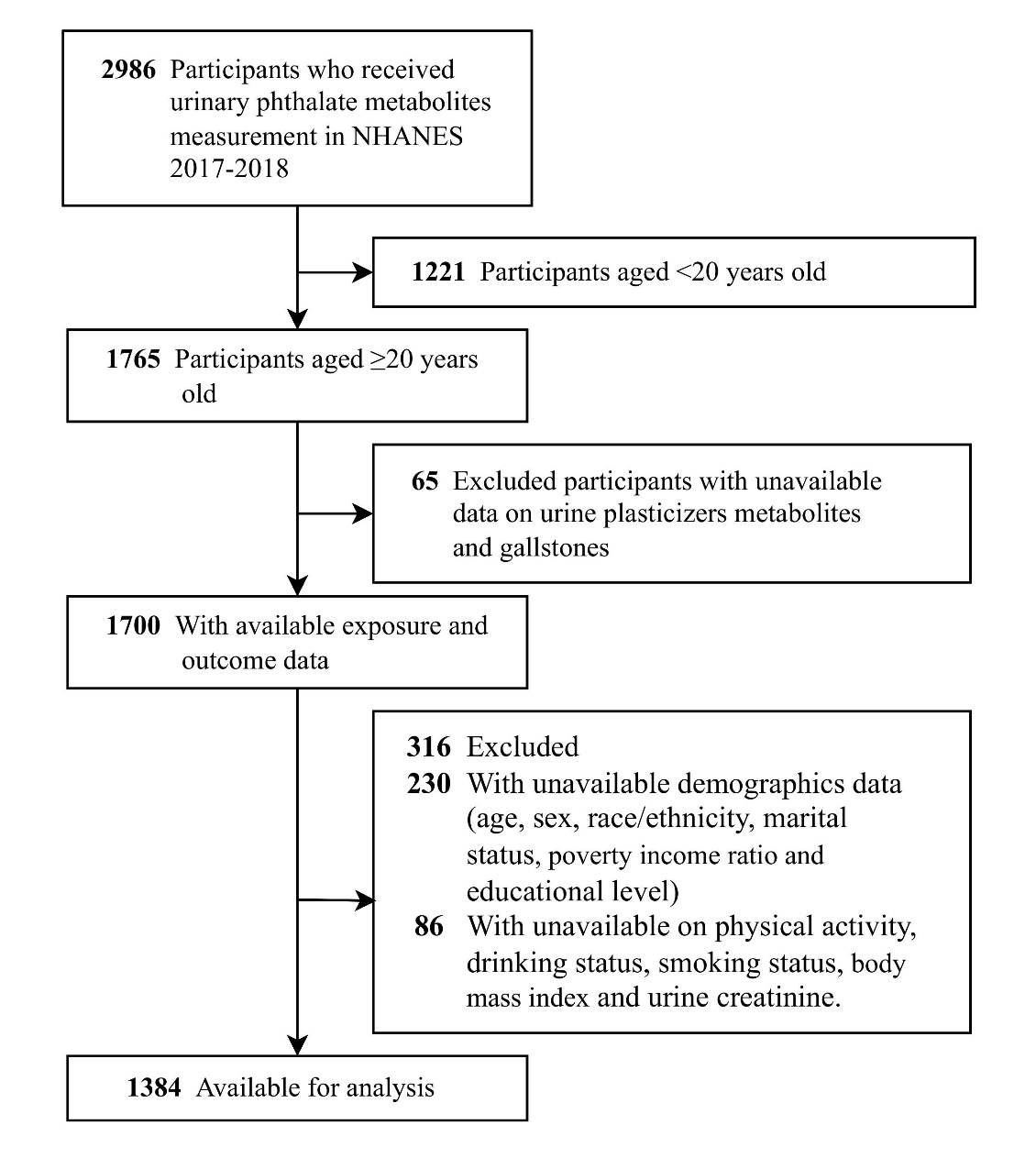


**Supplementary Figure 2. WQS model regression index weights for gallstone.**


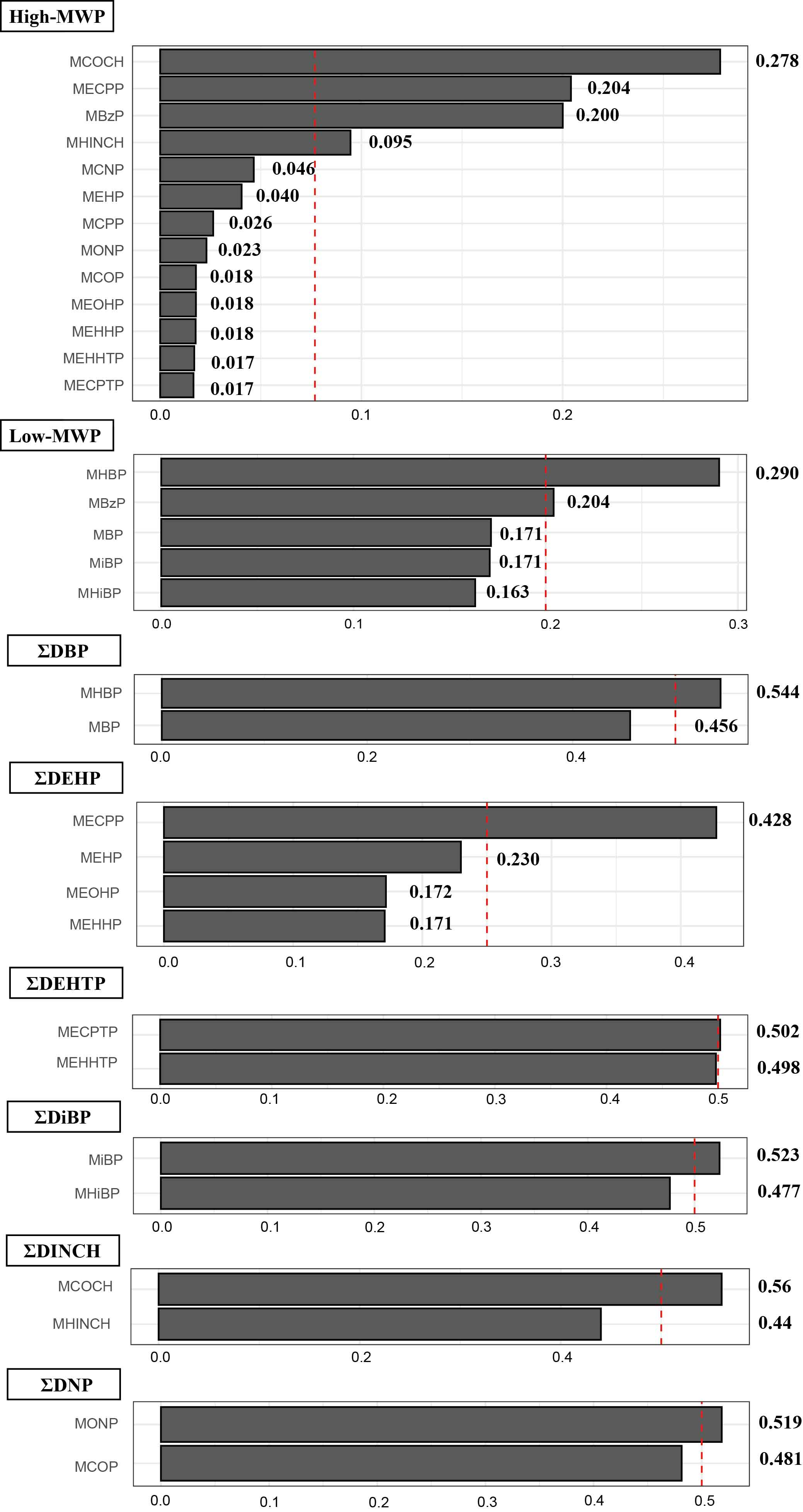


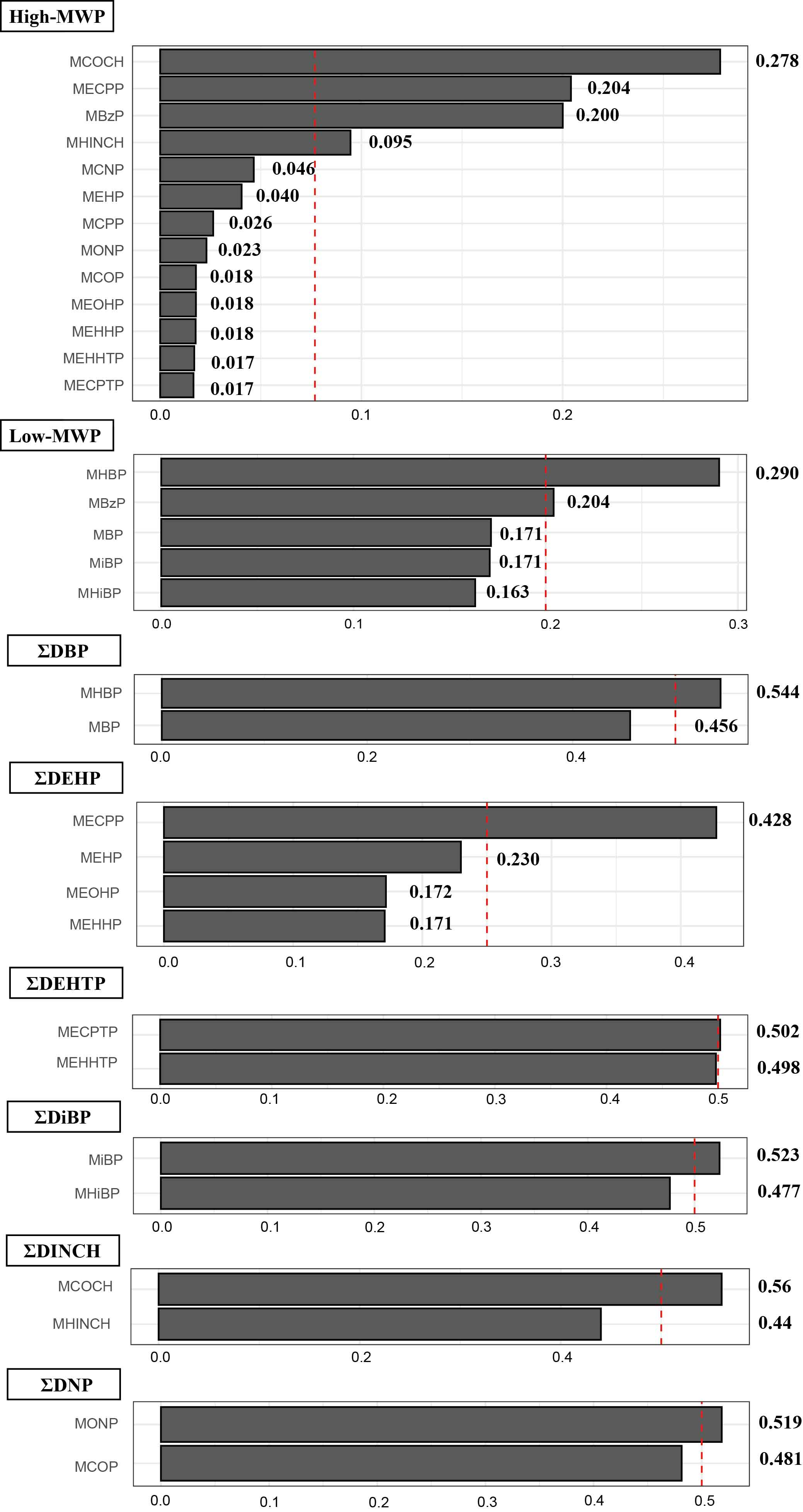


Models were adjusted for age, sex, race/ethnicity, poverty income ratio, marital status, education level, body mass index, physical activity, smoking and drinking status, and creatinine.

**Abbreviations:** DBP, di-n-butyl phthalate; DEHP, di(2-ethylhexyl) phthalate; DEHTP, di(2-ethylhexyl) terephthalate; DiBP, di-isobutyl phthalate; DINCH, 1,2-Cyclohexane dicarboxylic acid, diisononyl ester; DNP, di-isononyl phthalate; gWQS, generalized weighted quantile sum; High−MWP, high molecular-weight phthalate; Ln, natural logarithm; Low−MWP, low molecular-weight phthalate.

**Supplementary Figure 3. Association between individual phthalate metabolite and gallstone using restricted cubic splines regression.**


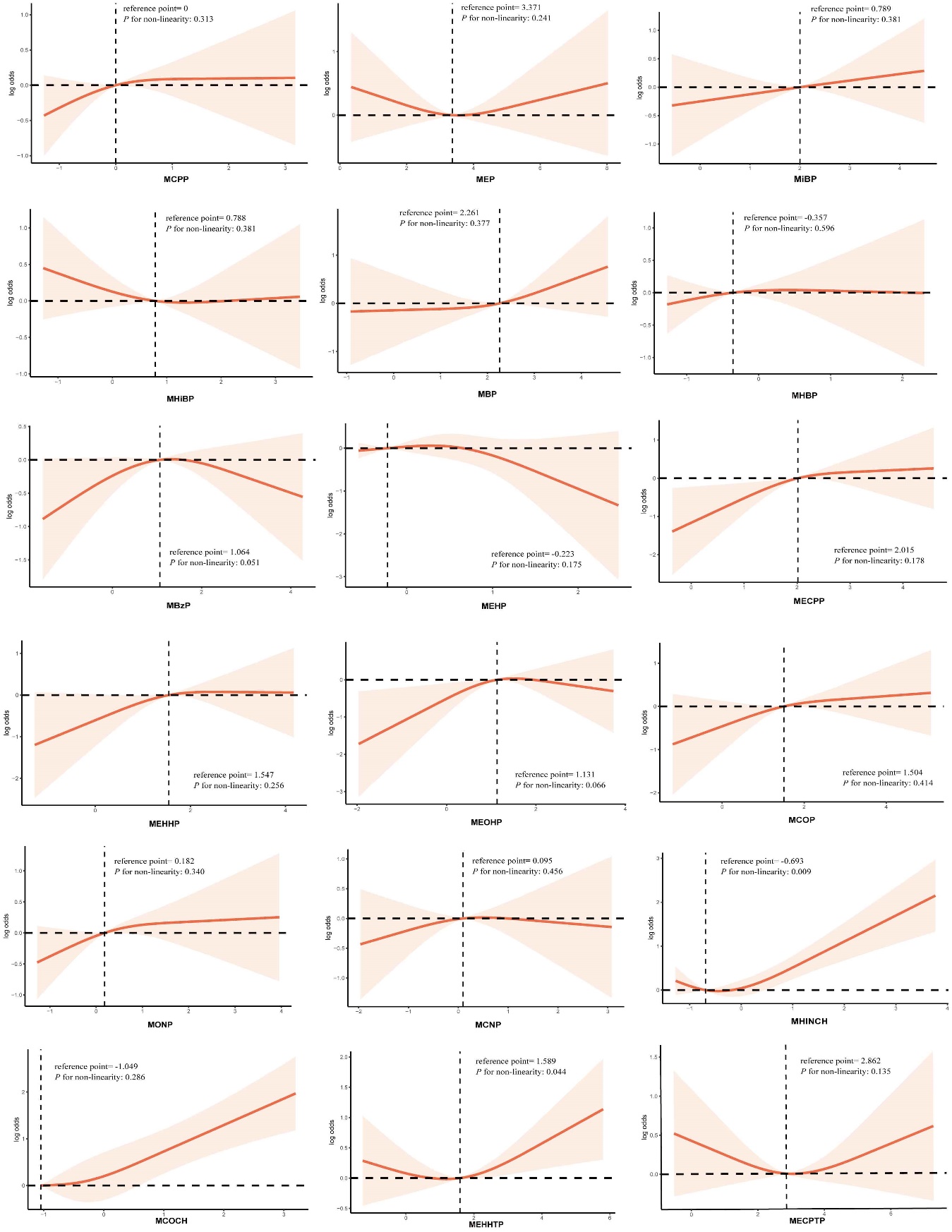


Models were adjusted for age, sex, race/ethnicity, poverty income ratio, marital status, education level, body mass index, physical activity, smoking and drinking status, and creatinine. **Abbreviations:** CI, confidence interval; Ln, natural logarithm; MBP, mono-n-butyl phthalate (ng/mL); MBzP, monobenzyl phthalate; MCNP, monocarboxy-isononyl phthalate; MCOCH, cyclohexane-1,2-dicarboxylic acid mono(carboxyoctyl) ester; MCOP, monocarboxyisooctyl phthalate; MCPP, mono (3-carboxypropyl) phthalate; MECPP, mono(2-ethyl-5-carboxypentyl) phthalate; MECPTP, mono-2-ethyl-5-carboxypentyl terephthalate; MEHP, mono(2-ethyl-5-carboxypentyl) phthalate; MEHHP, mono(2-ethyl-5-hydroxyhexyl) phthalate; MEHHTP, mono(2-ethyl-5-hydroxyhexyl) terephthalate; MEOHP, mono-(2-ethyl-5-oxohexyl) phthalate; MEP, mono-ethyl phthalate; MHBP, mono-3-hydroxybutyl phthalate; MHiBPP, mono-2-hydroxy-iso-butyl phthalate; MHINCH, cyclohexane-1,2-dicarboxylic acid mono(hydroxy-isononyl) ester; MiBP, Mono-isobutyl phthalate; MONP, mono-oxo-isononyl phthalate.

**Supplementary Figure 4. Association between sums phthalate metabolite and gallstone using restricted cubic splines regression.**


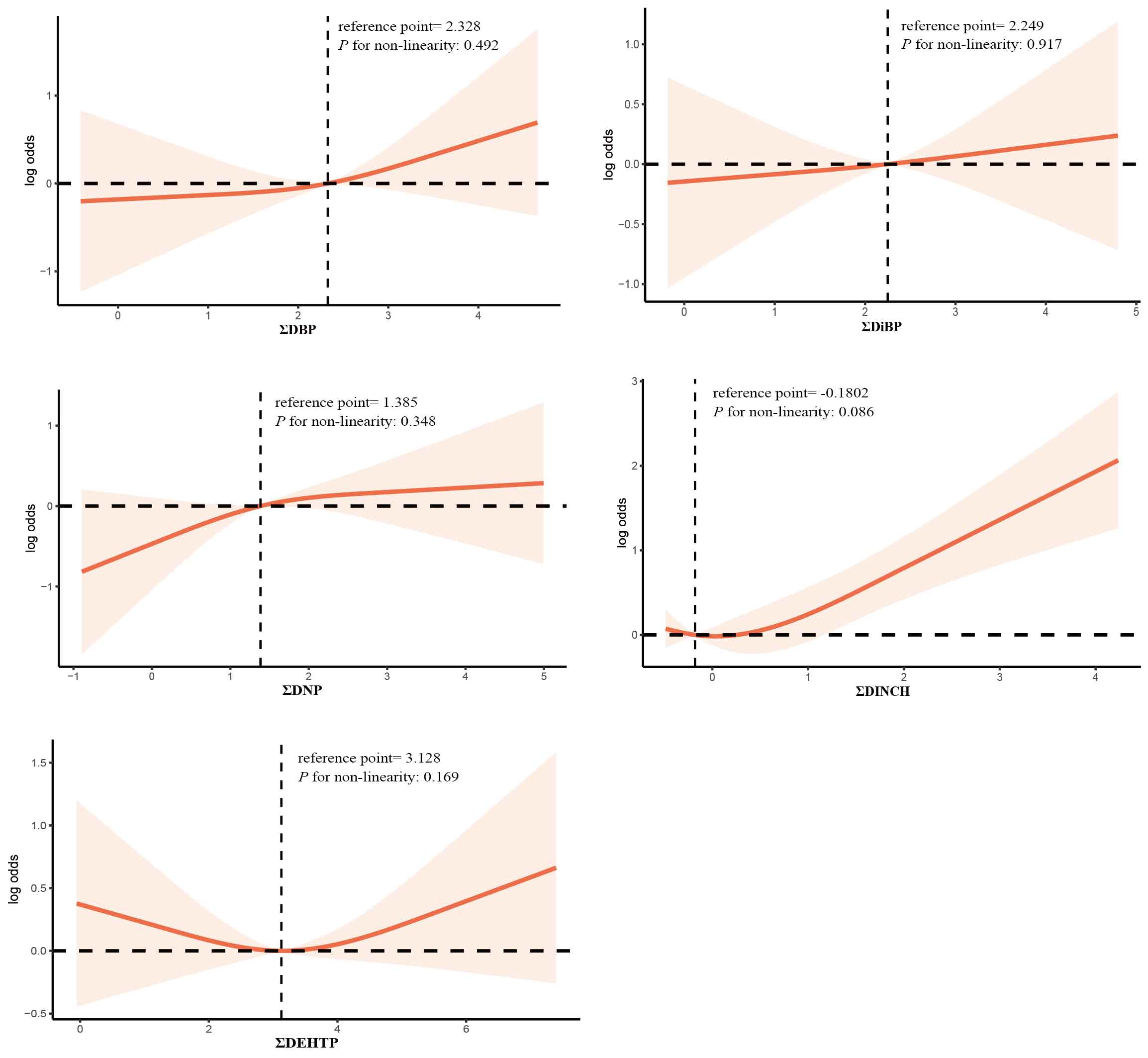


Models were adjusted for age, sex, race/ethnicity, poverty income ratio, marital status, education level, body mass index, physical activity, smoking and drinking status, and creatinine. **Abbreviations:** DBP, di-n-butyl phthalate; DEHP, di(2-ethylhexyl) phthalate; DEHTP, di(2-ethylhexyl) terephthalate; DiBP, di-isobutyl phthalate; DINCH, 1,2-Cyclohexane dicarboxylic acid, diisononyl ester; DNP, di-isononyl phthalate.

**Supplementary Figure 5. Posterior incorporation probabilities (PIPs) from the BKMR model.**


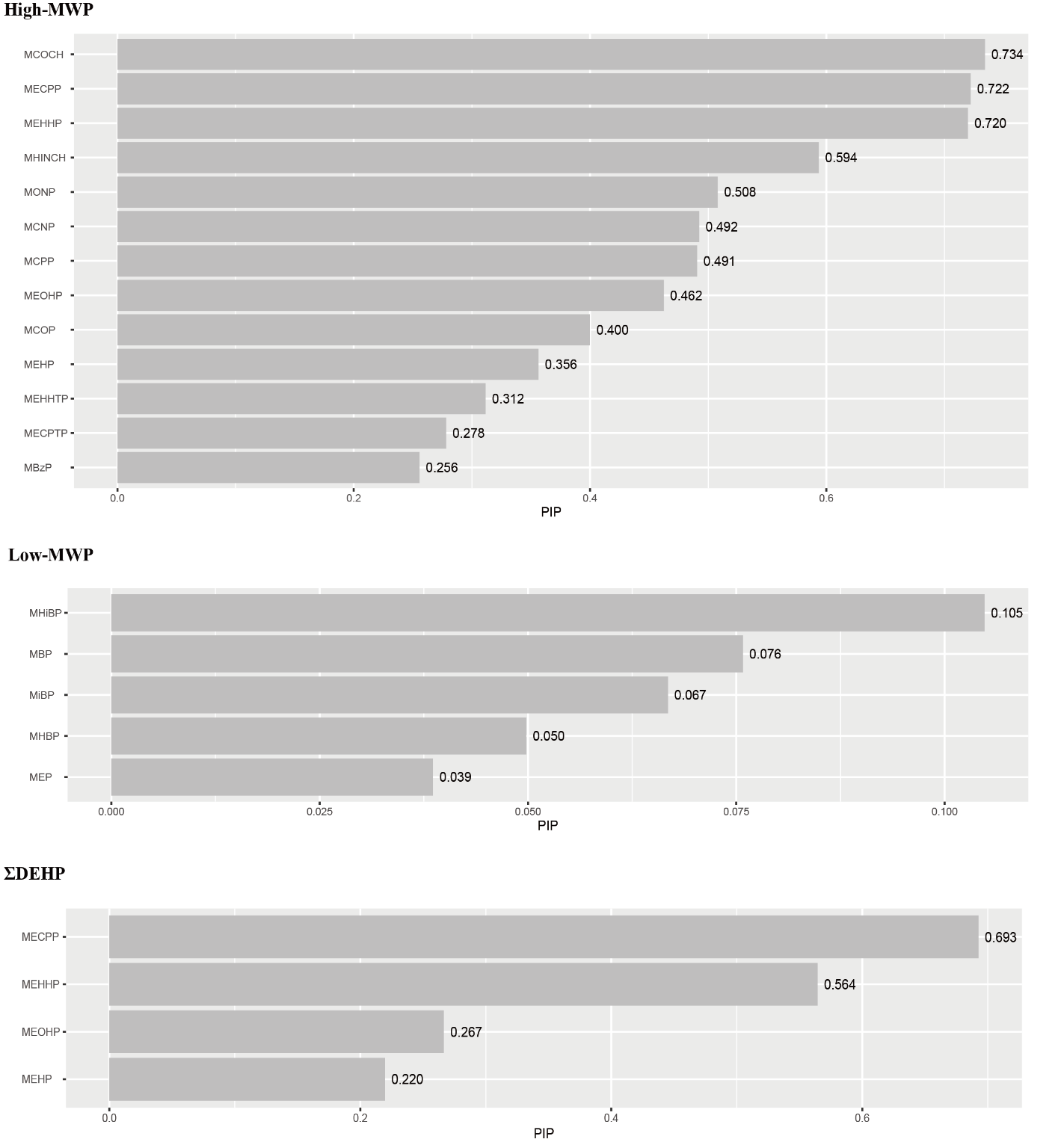


**Abbreviations:** BKMR, Bayesian kernel machine regression; DEHP, di(2-ethylhexyl) phthalate; High-MWP, high molecular-weight phthalate; Low-MWP, low molecular-weight phthalate.
